# Development of a Sensitive Outcome for Economical Drug Screening for Progressive Multiple Sclerosis Treatment

**DOI:** 10.3389/fneur.2016.00131

**Published:** 2016-08-15

**Authors:** Peter Kosa, Danish Ghazali, Makoto Tanigawa, Chris Barbour, Irene Cortese, William Kelley, Blake Snyder, Joan Ohayon, Kaylan Fenton, Tanya Lehky, Tianxia Wu, Mark Greenwood, Govind Nair, Bibiana Bielekova

**Affiliations:** ^1^Neuroimmunological Diseases Unit, National Institute of Neurological Diseases and Stroke, National Institute of Health, Bethesda, MD, USA; ^2^Department of Mathematical Sciences, Montana State University, Bozeman, MT, USA; ^3^Neuroimmunology Clinic, National Institute of Neurological Diseases and Stroke, National Institute of Health, Bethesda, MD, USA; ^4^EMG Section, National Institute of Neurological Diseases and Stroke, National Institute of Health, Bethesda, MD, USA; ^5^Clinical Trials Unit, National Institute of Neurological Diseases and Stroke, National Institute of Health, Bethesda, MD, USA

**Keywords:** multiple sclerosis, clinical trial, outcome measure, composite scale, progressive MS, disability scale, quantitative MRI

## Abstract

Therapeutic advance in progressive multiple sclerosis (MS) has been very slow. Based on the transformative role magnetic resonance imaging (MRI) contrast-enhancing lesions had on drug development for relapsing-remitting MS, we consider the lack of sensitive outcomes to be the greatest barrier for developing new treatments for progressive MS. The purpose of this study was to compare 58 prospectively acquired candidate outcomes in the real-world situation of progressive MS trials to select and validate the best-performing outcome. The 1-year pre-treatment period of adaptively designed IPPoMS (ClinicalTrials.gov #NCT00950248) and RIVITaLISe (ClinicalTrials.gov #NCT01212094) Phase II trials served to determine the primary outcome for the subsequent blinded treatment phase by comparing 8 clinical, 1 electrophysiological, 1 optical coherence tomography, 7 MRI volumetric, 9 quantitative T1 MRI, and 32 diffusion tensor imaging MRI outcomes. Fifteen outcomes demonstrated significant progression over 1 year (Δ) in the predetermined analysis and seven out of these were validated in two independent cohorts. Validated MRI outcomes had limited correlations with clinical scales, relatively poor signal-to-noise ratios (SNR) and recorded overlapping values between healthy subjects and MS patients with moderate-severe disability. Clinical measures correlated better, even though each reflects a somewhat different disability domain. Therefore, using machine-learning techniques, we developed a combinatorial weight-adjusted disability score (CombiWISE) that integrates four clinical scales: expanded disability status scale (EDSS), Scripps neurological rating scale, 25 foot walk and 9 hole peg test. CombiWISE outperformed all clinical scales (Δ = 9.10%; *p* = 0.0003) and all MRI outcomes. CombiWISE recorded no overlapping values between healthy subjects and disabled MS patients, had high SNR, and predicted changes in EDSS in a longitudinal assessment of 98 progressive MS patients and in a cross-sectional cohort of 303 untreated subjects. One point change in EDSS corresponds on average to 7.50 point change in CombiWISE with a standard error of 0.10. The novel validated clinical outcome, CombiWISE, outperforms the current broadly utilized MRI brain atrophy outcome and more than doubles sensitivity in detecting clinical deterioration in progressive MS in comparison to the scale traditionally used for regulatory approval, EDSS.

## Introduction

Therapeutic progress in relapsing-remitting multiple sclerosis (RRMS) has been facilitated by the recognition that contrast-enhancing lesions (CELs) on brain magnetic resonance imaging (MRI) can serve as a predictive marker of multiple sclerosis (MS) relapses. Utilizing this outcome allowed rapid, inexpensive screening of candidate agents. In contrast to RRMS, therapeutic development for progressive MS patients, who have few CELs and MS relapses, has been extremely slow. These patients relentlessly accumulate neurological disability, albeit at a pace that requires observation of hundreds of patients for a minimum of 2–3 years to reliably detect moderate (30–50%) therapeutic effects using the expanded disability status scale (EDSS) (1). Such studies utilize the majority of available patients and, therefore, allow screening of only a handful of therapeutic agents each decade.

Consequently, more sensitive outcomes are necessary to facilitate broader therapeutic advances for progressive MS. While quantitative MRI (qMRI) measures have been promoted as candidate outcomes (2, 3), a comprehensive comparison of qMRI markers with clinical outcomes and with each other is missing. Therefore, we integrated systematic comparisons of clinical, electrophysiological, optical coherence tomography (OCT) and a large number of qMRI measures as an adaptive part of the IPPoMS (double-blind, placebo-controlled Phase I/II clinical trial of Idebenone in patients with Primary Progressive Multiple Sclerosis; NCT00950248) and RIVITaLISe (Double Blind Combination of Rituximab by Intravenous and Intrathecal Injection Versus Placebo in Patients With Low-Inflammatory Secondary Progressive Multiple Sclerosis; NCT01212094) clinical trials and present the results.

## Materials and Methods

### Trial Design

IPPoMS and RIVITaLISe trials were randomized, double-blind, placebo-controlled trials with an adaptive design. The 2-year randomized treatment phase was preceded by a 1-year pre-treatment period, which served a dual purpose: (1) to determine a final primary outcome, by comparing 58 measures in the first ≥30 subjects, and to perform a new power analysis/sample size re-calculation using the selected outcome (this is the adaptive part of the design and represents the work described in this paper) and (2) to collect individualized disease-progression data while off therapy, as the baseline-versus-treatment paradigm is expected to enhance power (4). The default primary outcome for both trials was progression of brain atrophy measured by Structural Image Evaluation using Normalization of Atrophy (SIENA) methodology (5).

### Patient Population

Due to missing data, the first 35 primary progressive MS (PPMS; IPPOMS1 cohort) subjects who completed the IPPoMS pre-treatment baseline (before randomization into placebo or active treatment arm) were included to yield a minimum of 30 subjects per outcome, as defined in the protocol. The problem of missing clinical data at the beginning of the trial was solved by launching a database system in September 2013 that allows the principal investigator to confirm in real time that all measures were acquired according to the protocol. Some MRI data were missing because of technical issues with MRI acquisition (e.g., MRI machine problems). MRI data could also be missing if computer programs failed to run on the particular patient’s MRI, usually due to poor quality of the MRI caused by movement artifacts. The data loss in both instances should be considered missing completely at random.

The RIVITaLISe trial was recently terminated for futility, after interim analysis of the pharmacodynamic markers in the target organ showed that the pre-determined criteria for protocol continuation were not reached (6). Accrual of only 29 secondary progressive MS (SPMS) patients who completed the 1-year pre-treatment baseline prevented us from performing protocol-stipulated analysis of outcomes; therefore, we used this cohort (RIVITALISE cohort) as a validation cohort for the IPPOMS1 results. Finally, to avoid uncertainty as to whether SPMS and PPMS patients are comparable when it comes to clinical and MRI outcomes, we included all 34 remaining IPPOMS patients who completed the year-long pre-treatment baseline as of June 2015 and were not included in the IPPOMS1 as the second validation cohort (IPPOMS2 cohort).

Additionally, 7 healthy volunteers (HV) who served as technical controls and 303 untreated subjects (cross-sectional cohort), who presented for a work-up of putative neuro-immunological diseases, were recruited under a natural history protocol (Comprehensive Multimodal Analysis of Neuroimmunological Diseases of the Central Nervous System, NCT00794352), to validate correlations between the new CombiWISE metric and traditional clinical scales. Demographic data for all subjects are in Table 1.

**Table 1:** **Demographic data**.

		IPPOMS1 cohort	IPPOMS2 cohort	RIVITALISE cohort	Healthy volunteers	Cross-sectional cohort						
Diagnosis		*PPMS*	*PPMS*	*SPMS*	*HV*	*HV*	*NIND*	*OIND*	*CIS/RIS*	*RRMS*	*PPMS*	*SPMS*

*N* (F/M)		17/18	15/19	18/11	2/5	7/12	40/11	15/12	14/5	56/35	18/24	33/21

Age		55.0	55.7	54.2	41.4	36.6	49.7	46.0	38.4	40.6	53.3	54.7
	SD	7.8	7.1	7.4	13.1	11.1	10.0	15.8	13.8	10.9	9.4	7.9
	Range	36.7–65.7	36.0–70.3	38.3–65.0	23.7–64.7	23.5–56.7	27.6–70.4	18.5–70.7	20.7–60.3	18.0–68.6	29.8–74.7	38.3–69.4

DD		12.4	13.6	23.7	NA	NA	5.1	5.6	2.2	4.3	9.6	22.9
	SD	9.2	8.1	8.3	NA	NA	6.9	5.0	4.1	6.7	6.7	9.3
	Range	1.6–38.7	1.6–31.5	7.0–42.3	NA	NA	0.2–34.3	0.5–18.7	0.1–15.4	0.1–36.1	0.5–24.1	1.0–42.3

EDSS		5.6	5.0	6.1	0.3	0.4	2.3	3.3	1.1	1.8	4.6	5.9
	SD	1.3	1.6	0.8	0.5	0.5	1.9	2.4	0.8	1.4	1.7	1.2
	Range	2.0–6.5	2.0–6.5	2.5–6.5	0.0–1.0	0.0–1.0	0.0–6.5	0.0–8.0	0.0–2.5	0.0–6.5	1.5–6.5	2.0–7.0

SNRS		68.3	67.0	61.4	98.7	98.4	89.0	79.7	95.4	91.7	69.2	60.5
	SD	10.4	11.8	9.4	2.2	2.2	11.2	16.4	5.3	9.2	14.5	12.7
	Range	48–90	41–88	47.0–82.0	95–100	93–100	51–100	37–100	82–100	57–100	26–92	30–87

*F, female; M, male; N, number; DD, disease duration; SD, standard deviation; EDSS, Expanded Disability Status Scale; SNRS, Scripps Neurological Rating Scale; PPMS, primary progressive multiple sclerosis; HV, healthy volunteers; SPMS, secondary progressive multiple sclerosis; NIND, non-inflammatory neurological disorders; OIND, other inflammatory neurological disorders; CIS, clinically isolated syndrome, RIS, radiologically isolated syndrome, RRMS, relapsing-remitting multiple sclerosis. Age, DD, EDSS, and SNRS are provided as an average of the group. Age and DD are in years*.

### Inclusion Criteria

Eligible patients had clinically definite PPMS (IPPoMS trial) or SPMS (RIVITaLISe trial); aged 18–65 years (inclusive) with disability ranging from mild to moderate (EDSS 1–7, inclusive). Patients must not have received any immunomodulatory/immunosuppressive therapies for a period of at least 3 months prior to enrollment and must not have had any exposure to idebenone, coenzyme-Q10, or other mitochondrial-function promoting supplements more than three times the recommended daily dose for a period of at least 1 month before enrollment in the IPPoMS trial. Exclusion criteria included pregnancy, abnormal screening blood tests exceeding predefined limits, and/or clinically significant medical disorders that could expose the patient to undue risk or harm. A data safety monitoring board (DSMB) and institutional review board (IRB) approved a single patient exemption for a 70-year-old subject who otherwise fulfilled all inclusion criteria to be enrolled in the IPPoMS trial (Table 1).

Inclusion criteria for patients in the natural history protocol (cross-sectional cohort) were 18–75 years of age, presenting with a clinical syndrome consistent with immune-mediated central nervous system (CNS) disorder and/or neuroimaging evidence of inflammatory and/or demyelinating CNS disease. HV inclusion criteria were 18–75 years of age and vital signs within normal range at the time of screening visit. HV had to have no systemic disorder or CNS disease of any kind or other related risk factors.

### Study Oversight

All subjects provided written informed consent. The trials were approved by the Combined Neuroscience Institutional Review Board of the National Institutes of Health and relevant regulatory agencies. Monitoring was provided by Data and Safety Monitoring Boards.

### Pre-Defined Analysis of Outcomes

Because the SIENA methodology, the default primary trial outcome, calculated progression of brain atrophy as a percentage of baseline brain tissue, the same type of analysis was used for every other outcome; i.e., each biomarker quantified at month (Mo) 0 (before randomization) was expressed as a percentage of the Mo −12 value (considered to represent 100%). For each biomarker, we calculated a *z*-score as the average yearly change divided by the group standard deviation (SD). According to the protocol, *z*-scores (which are directly related to statistical power for a test of change) were designed to select the highest powered outcome. We observed a violation of the normality assumption in the analysis of some outcomes, questioning whether *z*-scores represented the best tool for outcome comparisons. As a compromise, in the IPPOMS1 cohort, we performed parametric statistical analysis after exclusion of outliers, without adjustment for multiple comparisons to obtain broad selection of candidate outcomes for validation. Outcomes from IPPOMS1 cohort at the 5% significance level were tested in two independent validation cohorts, RIVITALISE and IPPOMS2, using step-down Sidak (7) adjustments for multiple comparisons.

### Clinical Outcomes

EDSS (8); Scripps Neurological Rating Scale (SNRS) (9); all components of the Multiple Sclerosis Functional Composite (MSFC) (10), which includes 25 foot timed walk (25FW), 9 hole peg test (9HPT), and the Paced Auditory Serial Addition Test (PASAT); and the written Symbol Digit Modality Test (SDMT) were collected every 6 months.

### Electrophysiological Outcome

Single-pulse transcranial magnetic stimulation was performed using a Magstim 200. Motor evoked potentials were obtained using 130% resting threshold with mild activation of muscle. Central motor conduction time (CMCT) was calculated using the “F-wave method” (11). A total of six CMCTs were obtained: two from arms (at the abductor pollicis brevis) and four from legs (at the tibialis anterior and extensor digitorum brevis).

### Optical Coherence Tomography

Optical coherence tomography was obtained using the ZEISS Cirrus^(TM)^ HD-OCT Model 4000. The retinal nerve fiber layer (RNFL) thickness was quantified in four quadrants.

### MR Imaging

Magnetic resonance imaging of the brain was performed on a 3T Signa HDx (3TA; GE, Milwaukee, WI, USA) equipped with 16-channel head coil or on a 3T Skyra (3TD; Siemens, Malvern, PA, USA) with a 32-channel head coil. Follow-up MRIs were maintained on the same scanner as the first MRI. Seven HVs were scanned twice on each scanner to yield at least five technically adequate duplicates for test–retest reliability.

Magnetic resonance imaging of the brain on the 3TD included 3D-GRE with two flip angles for quantitative T1 (qT1) mapping [TR, 7.8 ms; TE, 3 ms; fractional anisotropy (FA), 3° and 18°; 1-mm isotropic resolution, TA 3.5 min each], and diffusion tensor imaging (DTI) (TR, 13000 ms; TE, 95 ms; *b*-value, 0 and 1000 s/mm^2^ in 2 repetitions of 30 directions; 2-mm isotropic resolution; acquisition time, 7.5 min). B1 correction for T1-mapping on the 3TD scanner was done with Bloch–Siegert-based B1 mapping (12). On the 3TA, the T1-mapping was done with SPGR sequences with two flip angles (TR, 7.8 ms; TE, 3 ms; FA, 3° and 17°; 1-mm isotropic resolution; acquisition time, 4 min each), and DTI (TR, 16000 ms; TE, 50 ms; *b*-value, 0 and 1100 s/mm^2^; 2.5-mm isotropic resolution; acquisition time, 9 min). B1 correction for T1-mapping on the 3TA was achieved using DESPOT HiFi technique (13) with two additional scans acquired with FSPGR sequence (FA of 3 and inversion time of 350 ms and 450 ms).

On each patient, additional clinical scans were acquired on the brain, including 3D-MPRAGE (TR, 3000 ms; TE, 3 ms; TI, 900 ms; FA 8°; 1-mm isotropic resolution, TA 6 min), 3D-FLAIR (TR, 4800 ms; TE, 354 ms; TI, 1800 ms; 1-mm isotropic resolution; acquisition time, 7 min), and PD/T2 (TR, 3540 ms; TE, 13 and 90 ms; 0.8-mm in-plane resolution; slice thickness, 2 mm; acquisition time, 4 min) on 3TD and 3D-FSPGR-BRAVO (TR, 1760 ms; TE, 3 ms; TI, 450 ms; FA 13°; 1-mm isotropic resolution; acquisition time, 5 min), 3D-FLAIR-CUBE (TR, 6000 ms; TE, 154 ms; TI, 1800 ms; 1-mm isotropic resolution; acquisition time, 9 min), and PD/T2 (TR, 5325 ms; TE, 20 and 120 ms; 1-mm in-plane resolution; slice thickness, 3 mm; acquisition time 4 min) on 3TA. If MRI contrast agent was administered, postgad T1-weighted images were acquired by repeating the 3D-GRE on the 3TD and 3D-FSPGE-BRAVO on the 3TA scanner.

The brain images were co-registered, skull-stripped, and tissue classified using LesionTOADS (14), and qT1-maps were created using JIST pipeline tools. DTI analysis was performed in three ways: (1) using TORTOISE (DTI_T_; https://science.nichd.nih.gov/confluence/display/nihpd), with diffusion images registered to the average co-registered T2 images from the two time points of each subject, (2) in the native space of the DTI acquisition (DTI_N_) with Eddy correction, and (3) with eddy-correct and non-linear registration to anatomical images (DTI_J_) in JIST using CATNAP and RESTORE tools (15, 16). Subject-specific regions of interest (ROIs) were drawn on the co-registered 3D-MPRAGE or 3D-FSPGR-BRAVO images for volume, qT1, and DTI_J_ measurements (Figure S1 in Supplementary Material), and on the DTI (Figure S2 in Supplementary Material) images for the DTI_N_ and DTI_T_ analysis. Fourteen ROIs [two caudate, two putamen, four internal capsules (two anterior and two posterior limbs), two thalamus, midbrain, pons, medulla, and corpus callosum] were drawn in the brain. For symmetrical structures, ROIs in each hemisphere were analyzed as a single structure. ROIs were eroded (by two pixels for the majority of structures) in MIPAV to limit partial volume averaging. The ROIs drawn on the brain were copied on to the co-registered qT1-maps as well as co-registered tensor images to derive average quantitative values.

Percent change in brain volume was calculated using SIENA (V-SIENA (5); http://fsl.fmrib.ox.ac.uk/fsl), while volumes of brain (V-Brain), ventricles (V-Ventricles), cortical gray matter (V-CorticalGM) and thalamus (V-Thalamus) were calculated using LesionTOADS tissue segmentation. A cross-sectional area of the upper cervical SC at the level of Dens (A-CS-Dens) was calculated from manually drawn ROIs on individual GRE or SPGR images (using OSIRIX and MIPAV).

### Data Collection

The EDSS and SNRS were performed by the same clinician, who had no knowledge/intervention in collecting any other outcomes. 9HPT, 25FW, PASAT, and SDMT were performed by non-clinical investigators, who had no knowledge/intervention in collecting other outcomes. MRI analyses were performed by another set of non-clinical personnel, who had no knowledge/intervention in collecting clinical outcomes.

### Development of Combinatorial Weight-Adjusted Disability Score

To mathematically optimize the new scale (i.e., CombiWISE) with relative weights of different subscales that are not distorted by individual observations, each clinical scale was re-scaled by its maximum achievable value so that all values lie between 0 and 1 making the different scales directly comparable. The three longitudinal cohorts of progressive MS patients (IPPOMS1, IPPOMS2, and RIVITALISE) were combined and the subjects were then randomly permuted multiple times between training and validation datasets with 70% of the subjects allocated to each training dataset. In order to efficiently estimate the contributions of the failure to complete 9HPT or 25FW, the randomization was constrained to balance the number of subjects in each training and validation dataset with failed attempts on non-dominant hand 9HPT (NDH-9HPT), dominant hand 9HPT (DH-9HPT), 25FW, and a combination of NDH-9HPT and 25FW. For each of the constructed training datasets, a genetic algorithm (GA) implemented in the *GA* package (17) in R (18) was used to construct a linear combination of EDSS, SNRS, log25FW (log2 of average of two attempts on 25FW, or 0 if at least one trial was unsuccessful), 25FW_FAIL_ (1 if patient failed either attempt on 25FW; otherwise 0), logNDH-9HPT (log2 of average of two attempts on 9HPT with non-dominant hand; otherwise 0), NDH_FAIL_ (1 if patient failed either attempt on 9HPT with non-dominant hand; otherwise 0), logDH-9HPT (log2 of average of two attempts on 9HPT with dominant hand; otherwise 0), DH_FAIL_ (1 if patient failed either attempt on 9HPT with dominant hand, otherwise 0), PASAT, and SDMT that maximizes the evidence of a change over time (test statistic) from a linear mixed model (19) estimated using the *nlme* package (20). The model contained a random intercept for each subject (in order to account for three repeated measures on each subject; Mo −12, Mo −6, and Mo 0) and assumes a linear change over these times. The sign of the individual weights were constrained to be the direction of disease progression (i.e., positive for EDSS since higher values of EDSS indicate more progression, negative for SNRS since smaller values of SNRS indicate more progression). The scale was optimized for 200 permutations, followed by dropping of four variables with weights routinely close to 0 (logDH-9HPT, DH_FAIL_, PASAT, and SDMT). The final weights after removing these unused variables were generated as an average of the selected weights from 500 permutations of the training data set (referred to hereafter as relative weights that allow comparison of overall contributions of individual components to the developed scale), followed by their linear re-scaling to generate the CombiWISE scale that ranges from 0 to 100 (referred hereafter as computing weights that allow for construction of a scale with range specified above, with higher values indicating more disease severity). The performance of CombiWISE against traditional clinical outcomes was measured in 500 permutations of the retained validation data sets. The R code for the development of the CombiWISE scale is available in Data S1 in the Supplementary Material. Table S1 in the Supplementary Material provides an embedded formula that calculates CombiWISE values based on entered EDSS, SNRS, 25FW, and 9HPT-NDH.

### Statistical Methods

For each biomarker, the relative percentage change over 1 year (= 100 × (score at period 0 − score at period −12)/score at period −12) was calculated and used as an outcome measure. For most biomarkers, the relative change had normal or near-normal (both kurtosis and skewness between −1 and 1) distributions (based on Shapiro–Wilk test) after excluding a few extreme outliers [<Q1-3IQR, or >Q3+3IQR, where Q1 and Q3 are the first and third quartiles and IQR is the interquartile range (Q3−Q1)]. One-sample *t*-tests were performed to test the null hypothesis: μ = 0 (the mean relative change equals to 0) for each biomarker in the IPPOMS1 cohort. The same test was performed for IPPOMS2 and RIVITALISE cohorts separately for any biomarkers with *p* < 0.05 in IPPOMS1, with each set of *p*-values corrected for multiple testing using step-down Sidak method (7).

The power analysis was performed for both a two-group parallel design and a one-group baseline-versus-treatment design. Since IPPoMS is a 2-year randomized controlled trial, for each biomarker, the relative change in 2 years [= 100 × (measure score in 2 year − baseline)/baseline] was used as an outcome measure, which was assumed to follow a normal distribution, has changed linearly over time and had homogeneity of variance. The drug was assumed to have 50%, 40%, or 30% effect. The mean and SD from the observed relative change in the first year without treatment was used for the calculations.

For a two-group design, a two-tailed two-sample *t*-test was used to test the null hypothesis: μ_1_ = μ_2_, where μ_1_ is placebo group mean change and μ_2_ is treated group mean change. For example, for an outcome variable with 10% measured relative change in the 1-year pre-treatment period, the placebo group mean was expected to change 20% over 2 years of treatment, and the treated group mean was expected to change 10% (12% and 14%) if the drug had 50% (40% and 30%, respectively) efficacy.

For the one-group design, two-tailed one-sample *t*-test was used to test the null hypothesis: μ = μ_0_, where μ_0_ is null hypothesis mean and μ is the alternative hypothesis mean, with analogously estimated 50% (40% and 30%) drug effects.

For each biomarker, the sample sizes were estimated to reject the null hypotheses with 80% power at the 5% significance level (not adjusted for multiple outcomes) for the two designs.

The individual biomarker and power analyses were performed using SAS 9.2 and GraphPad Prism 6 software.

## Results

The results for all 58 measured variables are summarized in Table 2.

**Table 2:** **Statistical information for all measured variables**.

Category (No. of measures)	Biomarker abbreviation	Biomarker description	IPPOMS1				IPPOMS2	RIVITALISE
			Mean% Δ	SD	*z*-score^a^	*p*-value Δ (*t*-test)^b^	*p*-value Δ (*t*-test)^c^	*p*-value Δ (*t*-test)^c^
Clinical (8)	EDSS	Expanded Disability Status Scale	6.48	14.15	0.4580	**0.0116**	**0.0109**	0.3293^d^
	SNRS	Scripps Neurological Rating Scale	−4.54	8.71	0.5212	**0.0046**	**0.0052**	0.0766
	25FW	25 Foot Walk (average of two attempts)	64.52	193.12	0.3341	**0.0029**	0.1537	0.3293
	9HPT	9 Hole Peg test (average of two attempts per hand)	18.73	86.89	0.2156	0.1341		
	PASAT	Paces Auditory Serial Addition Test	5.72	23.88	0.2395	0.3438		
	MSFC	MS functional composite (Composite of 25FW, 9HPT, and PASAT)	−11.24	277.32	0.0405	0.3197		
	SDMT	Symbol Digit Modality Test (# correct)	−2.26	12.13	0.1863	0.2930		
	CombiWISE	Composite of EDSS, SNRS, 25FW, and 9HPT	9.10	13.23	0.6878	**0.0003**	**0.0107**	**0.0445**
Electrophysiological (1)	Σ CMCT	Sum of 6 central motor conduction times, one per each upper and two per each lower extremity	5.07	21.18	0.2394	0.1852		
Imaging-volumetric (7)	V-SIENA	Change in brain volume (SIENA method)	−0.70	1.70	0.4118	**0.0178**	0.0980	0.6454
	V-Brain	Brain volume (LesionTOADS method)	−0.12	2.17	0.0553	0.9803		
	V-Ventricles	Ventricular volume (LesionTOADS method)	3.45	7.01	0.4922	**0.0081**	**0.0032**	**0.0249**
	V-CorticalGM	Volume of cortical gray matter (LesionTOADS method)	1.26	15.71	0.0802	0.4792		
	V-Thalamus	Volume of thalamus (LesionTOADS method)	−3.84	8.86	0.4334	**0.0084**	0.4416	0.4314
	V-Caudate + Putamen	Volume of caudate and putamen (LesionTOADS method)	−1.61	13.65	0.1179	0.3879		
	A-CS-Dens	Cross-sectional area of spinal cord (dens level)	−0.31	23.04	0.0135	0.9418		
Imaging-OCT (1)	OCT	Optical coherence tomography	−1.48	4.72	0.3136	0.0970		
Imaging-qT1 (9)	qT1-IC	qT1 of anterior limb of internal capsule	1.71	4.84	0.3533	0.1155		
	qT1-CC	qT1 of corpus callosum	1.21	5.64	0.2145	0.2507		
	qT1-Caudate	qT1 of the head of the caudate nucleus	1.71	5.84	0.2928	0.2369		
	qT1-Putamen	qT1 of putamen	0.99	5.47	0.1810	0.3304		
	qT1-Thalamus	qT1 of thalamus	2.09	5.00	0.4180	**0.0296**	0.6820	0.4314
	qT1-Midbrain	qT1 of midbrain (axial section)	0.93	5.22	0.1782	0.3394		
	qT1-Pons	qT1 of pons (sagittal section)	0.00	5.06	0.0000	0.9975		
	qT1-Medulla	qT1 of medulla (axial section)	0.19	5.95	0.0319	0.8598		
	qT1-SC-Dens	qT1 of spinal cord (dens level; axial section)	1.46	31.53	0.0463	0.1655		
Imaging-DTI Tortoise (32)	DTI-T-IC	Radial diffusivity of internal capsule (anterior)	0.78	9.56	0.0816	0.6584		
	DTI-II-IC	Axial diffusivity of internal capsule (anterior)	3.69	12.19	0.3027	0.1455		
	DTI-MD-IC	Mean diffusivity of internal capsule (anterior)	2.23	6.85	0.3255	0.0853		
	DTI-FA-IC	Fractional anisotropy of internal capsule (anterior)	2.47	11.26	0.2194	0.4937		
	DTI-T-ICPost	Radial diffusivity of internal capsule (posterior)	1.30	11.15	0.1166	0.5279		
	DTI-II-ICPost	Axial diffusivity of internal capsule (posterior)	2.66	8.68	0.3065	0.1064		
	DTI-MD-ICPost	Mean diffusivity of internal capsule (posterior)	1.89	6.25	0.3024	0.1080		
	DTI-FA-ICPost	Fractional anisotropy of internal capsule (posterior)	1.24	7.12	0.1742	0.7887		
	DTI-T-CC	Radial diffusivity of corpus callosum	−1.77	14.81	0.1195	0.5172		
	DTI-II-CC	Axial diffusivity of corpus callosum	1.69	7.51	0.2250	0.4837		
	DTI-MD-CC	Mean diffusivity of corpus callosum	0.59	7.12	0.0829	0.6513		
	DTI-FA-CC	Fractional anisotropy of corpus callosum	1.40	4.15	0.3373	0.1439		
	DTI-T-Caudate	Radial diffusivity of the head of the caudate	4.78	8.89	0.5377	**0.0063**	**0.0032**	0.0783
	DTI-II-Caudate	Axial diffusivity of the head of the caudate	3.14	7.52	0.4176	**0.0294**	**0.0035**	**0.0342**
	DTI-MD-Caudate	Mean diffusivity of the head of the caudate	4.06	7.72	0.5259	**0.0074**	**0.0032**	**0.0457**
	DTI-FA-Caudate	Fractional anisotropy of the head of the caudate	−2.48	14.25	0.1740	0.3480		
	DTI-T-Putamen	Radial diffusivity of the putamen	0.92	7.16	0.1285	0.9322		
	DTI-II-Putamen	Axial diffusivity of the putamen	2.18	6.59	0.3308	0.0810		
	DTI-MD-Putamen	Mean diffusivity of the putamen	1.40	6.62	0.2115	0.5137		
	DTI-FA-Putamen	Fractional anisotropy of the putamen	7.27	18.92	0.3842	**0.0443**	**0.0052**	0.6454
	DTI-T-Thalamus	Radial diffusivity of the thalamus	1.16	5.64	0.2057	0.2674		
	DTI-II-Thalamus	Axial diffusivity of the thalamus	1.63	6.22	0.2621	0.1608		
	DTI-MD-Thalamus	Mean diffusivity of the thalamus	1.35	5.40	0.2500	0.1817		
	DTI-FA-Thalamus	Fractional anisotropy of the thalamus	2.21	11.16	0.1980	0.2874		
	DTI-T-Midbrain	Radial diffusivity of the midbrain (axial section)	3.17	10.42	0.3042	0.1063		
	DTI-II-Midbrain	Axial diffusivity of the midbrain (axial section)	3.40	10.91	0.3116	0.1558		
	DTI-MD-Midbrain	Mean diffusivity of the midbrain (axial section)	3.01	7.99	0.3767	**0.0484**	**0.0433**	**0.0018**
	DTI-FA-Midbrain	Fractional anisotropy of the midbrain (axial section)	1.60	11.73	0.1364	0.8951		
	DTI-T-Medulla	Radial diffusivity of the medulla (axial section)	6.58	10.61	0.6202	**0.0020**	**0.0034**	**0.0157**
	DTI-II-Medulla	Axial diffusivity of the medulla (axial section)	2.13	7.16	0.2975	0.1132		
	DTI-MD-Medulla	Mean diffusivity of the medulla (axial section)	4.46	8.17	0.5459	**0.0057**	**0.0032**	**0.0138**
	DTI-FA-Medulla	Fractional anisotropy of the medulla (axial section)	−5.19	14.96	0.3469	0.0673		

*One-sample t-test was used to calculate p-values for the tests for the yearly change for each variable. P-values in bold highlight variables that showed significant at the 5% level*.

*^a^z-score was calculated by dividing the mean group change over 1 year by the group SD*.

*^b^Raw p-values are displayed*.

*^c^Only variables that showed statistical significance in IPPOMS1 cohort were subjected to statistical testing in IPPOMS2 and RIVITALISE cohorts. p-values are adjusted for tests performed on 15 variables using step-down Sidak method*.

*^d^t-test performed on the whole dataset – outlier removal would eliminate 32% of subjects from the cohort*.

### Standard Clinical Scales

Both EDSS [Δ = 6.48% (SD = 14.15%); *p* = 0.0116] and SNRS [Δ = −4.54% (8.71); *p* = 0.0046] demonstrated statistically significant progression, with SNRS having a higher *z*-score (0.52 vs. 0.46; Table 2). 25FW also showed statistically significant progression over the period of 1 year [Δ = 64.52% (SD = 193.12%); *p* = 0.0029]. MSFC or its remaining individual components (i.e., 9HPT and PASAT) did not show any evidence of a significant change between Mo −12 and Mo 0. Similarly, SDMT did not show significant yearly progression.

### Electrophysiological Measures

In order to calculate one electrophysiological outcome, we have added six CMCT values to create a composite Σ CMCT measure. The group change over 1 year was not statistically significant [Δ = 5.07% (21.18); *p* = 0.1852].

### MRI Volumetric Outcomes

While change in the brain volume measured by LesionTOADS (V-Brain) was not statistically significant, the percent change in brain volume calculated by SIENA was significantly reduced over 1 year [V-SIENA; Δ = −0.70% (1.70); *p* = 0.0178] with a *z*-score of 0.41. Brain tissue segmentation revealed no evidence of a change in cortical gray matter (V-CorticalGM), caudate and putamen, but statistically significant decrease in thalamic volume [V-Thalamus Δ = −3.84% (8.86); *p* = 0.0084] and enlargement of ventricles [V-Ventricles; Δ = 3.45% (7.01); *p* = 0.0081]. Both of these segmented volumetric outcomes achieved higher *z*-scores than whole brain atrophy (V-SIENA).

None of the cross-sectional areas of the upper cervical SC changed significantly over 1 year; therefore, we only highlight the best-performing of these outcomes, measured at the level of dens (C1).

### Optical Coherence Tomography

Because the temporal quadrant of retinal nerve fiber layer has the most-pronounced thinning in MS (21), we have used the sum of the temporal quadrants from both eyes as a single OCT measure. This biomarker did not show evidence of progression over 1 year.

### MRI Tissue Integrity Measures: qT1

Only one of the nine qT1 biomarkers, measured in manually segmented ROIs corresponding to thalamus (qT1-Thalamus) showed statistically significant change over 1 year [Δ = 2.09% (5.00); *p* = 0.0296].

### MRI Tissue Integrity Measures: DTI

Initially, we used the identical co-registration method (i.e., JIST; DTI_J_) for qT1 and DTI measures of CNS tissue integrity. However, DTI_J_ data demonstrated unacceptably high scan-rescan variability in HVs (at times > 100%), which led us to re-analyze DTI scans using two different technologies: (1) we drew separate ROIs for Mo −12 and Mo 0 on un-registered native DTI scans (DTI_N_) and (2) we co-registered DTI scans to anatomical images using TORTOISE algorithm (DTI_T_). The DTI_T_ method outperformed DTI_N_ for all DTI biomarkers and, therefore, only DTI_T_ data are presented.

Seven DTI outcomes measured in four brain structures changed significantly over 1 year in the IPPOMS1 cohort: radial- [Δ = 4.78% (8.89); *p* = 0.0063], axial- [Δ = 3.14% (7.52); *p* = 0.0294], and mean diffusivity (MD) of the head of the caudate nucleus [Δ = 4.06% (7.72); *p* = 0.0074], FA of the putamen [Δ = 7.27% (18.92); *p* = 0.0443], MD of the midbrain [Δ = 3.01% (7.99); *p* = 0.0484] and the radial- [Δ = 6.58% (10.61); *p* = 0.0020] and MD of the medulla [Δ = 4.46% (8.17); *p* = 0.0057].

### Validation of Biomarkers that Reached Statistical Significance in the IPPOMS1 Cohort in Two Additional Longitudinal Progressive MS Cohorts (RIVITALISE and IPPOMS2)

Fifteen outcomes that reached statistical significance based on unadjusted *p*-values in the IPPOMS1 cohort were evaluated for statistically significant progression over 1 year in two independent validation cohorts consisting of PPMS (IPPOMS2) and SPMS (RIVITALISE) patients with identical inclusion criteria. Eleven outcomes also showed statistical significance in the IPPOMS2 cohort after adjustment for multiple comparisons, but only six outcomes validated in the smaller RIVITALISE cohort (Table 2).

From clinical measures, SNRS barely missed the cut-off for statistical significance in the RIVITALISE cohort. From MRI volumetric measures, only ventricular volume (V-Ventricles) validated in both cohorts. Finally, from MRI measures of CNS tissue integrity, MD was the most successful DTI biomarker and it validated significant progression when measured in three ROIs: the head of the caudate nuclei, midbrain, and medulla. Additionally, radial diffusivity of the medulla and axial diffusivity of caudate nuclei also demonstrated statistically significant change in both validation cohorts.

### Correlations between Validated Outcomes and EDSS

**Figure 1:**
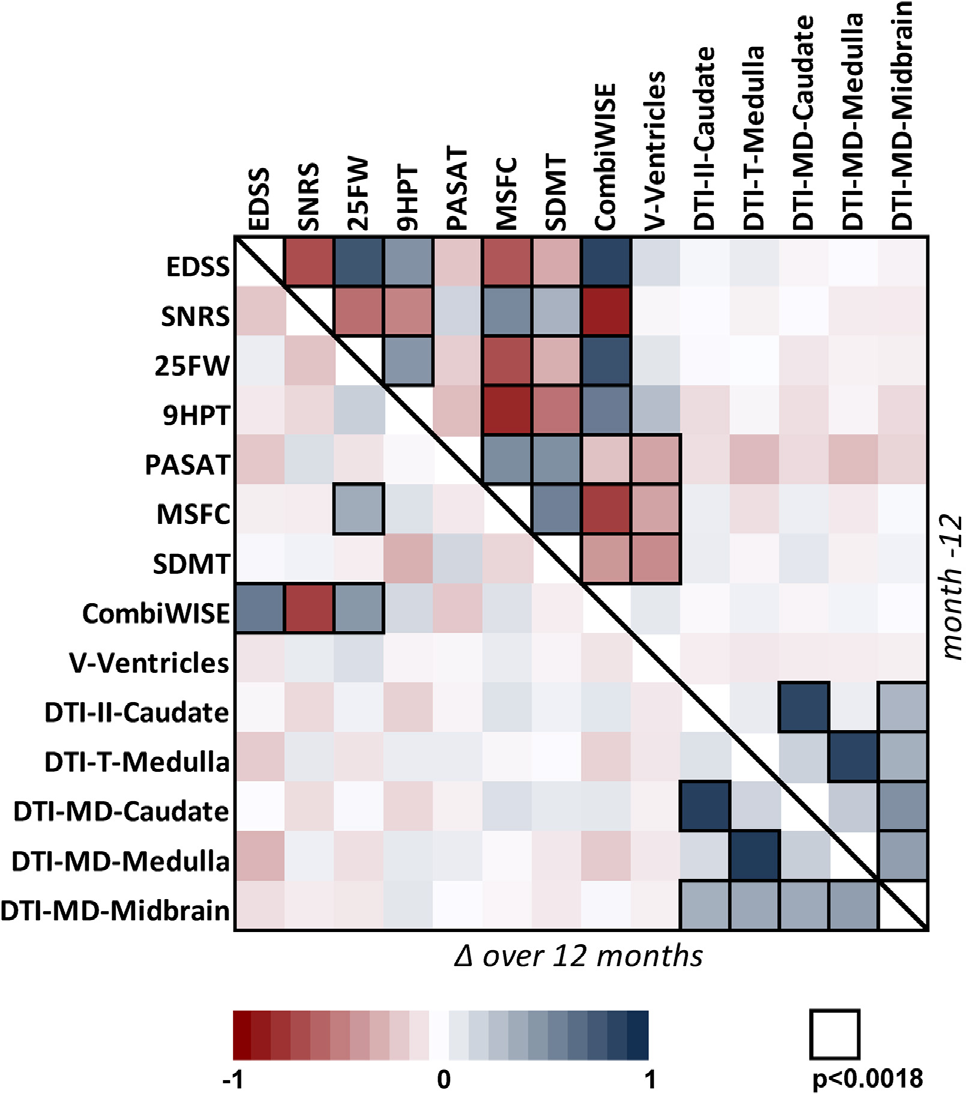
**Correlation matrix for 14 clinical and MRI measures in the cohort of 98 progressive MS patients**. Correlation matrix for Mo −12 cross-sectional data (above the diagonal) and relative percentage change (Δ) over 1 year (below the diagonal) in the progressive MS cohort. The heatmap shows positive (shades of blue) and negative (shades of red) Spearman correlations. Black border of a window indicates Spearman correlation with *p* < 0.0018 (the Bonferroni-adjusted significance level for multiple comparisons of 28 tested variables). For exact values of Spearman correlation coefficients, *p*-values, and number observations, see Table S2 in Supplementary Material.

Because clinical progression as measured by EDSS has been an accepted outcome for regulatory approval of MS treatments, we evaluated correlations between validated outcomes and EDSS, in cross-sectional (i.e., including Mo −12 data for 98 progressive MS patients from all three longitudinal cohorts) and longitudinal (i.e., correlations between yearly changes measured in identical 98 patients) paradigms. We also included other clinical scales in the correlation matrix for instructive purposes [Figure 1 (exact correlation coefficients, *p*-values, and number of observations are in Table S2 in Supplementary Material)].

In the cross-sectional paradigm, we observed strong to moderate correlations between all clinical outcomes that had small *p*-values, with exception of the cognitive test PASAT, which was only moderately correlated with another cognitive test SDMT. We also observed statistically significant correlations of relatively mild strength between ventricular volume and clinical biomarkers that capture cognition and fine finger movements/coordination (i.e., SDMT, MSFC, PASAT, and 9HPT), but not with EDSS. Finally, DTI measures were generally correlated with each other, but not with any clinical outcome.

As expected, fewer correlations were observed in the longitudinal paradigm: CombiWISE (see below) was the only scale that showed strong, statistically significant correlation of its yearly change with three out of four clinical outcomes (EDSS, SNRS, and 25FW) that contribute to its computation. By contrast, yearly change in MSFC, another composite scale, demonstrated significant, but weak correlation with only one (25FW) of its three components. Again, strong correlations were observed between different DTI measures. However, no statistically significant correlation was observed between MRI measures and clinical scales in the longitudinal paradigm.

### Composite Clinical Score: CombiWISE

The discrepancy between strong correlations among clinical scales in the cross-sectional paradigm and the lack of correlations in the 1 year longitudinal study indicate that like other outcomes, tested clinical scales suffer from low sensitivity confounded by measurement noise. Repeated measurements can enhance signal-to-noise ratio (SNR). To the extent to which clinical scales capture overlapping elements of disability, they represent a form of repeated measures. For example, slight worsening in one clinical score but improvement in another may reflect performance noise rather than true disability. A structural substrate to observed change is expected to affect overlapping domains of several clinical scores congruently. Thus, using a combination of clinical scales with overlapping elements amplifies the true disability and limits measurement noise. However, differences in *z*-scores also indicate that clinical scales differ in sensitivity and specificity. Therefore, combination of clinical scales should be based on their measured performance, giving a greater weight to the measures that have higher sensitivity and lower measurement noise. We tested this hypothesis by first constructing a conceptual model of the combinatorial weight-adjusted disability score (CombiWISE v.0; see Figure S3 in Supplementary Material for details) based on the collected clinical data exclusively in the IPPOMS1 cohort. We then validated its sensitivity for longitudinal change and superiority against other clinical measures in IPPOMS2 and RIVITALISE cohorts (Figure S3 in Supplementary Material). Because CombiWISE v.0 represented only one possible model from measured data, we next employed statistical modeling using a GA (22–24) to numerically optimize CombiWISE for its ability to detect yearly changes across a suite of random permutations of the acquired data from all 98 progressive MS patients (see Materials and Methods).

**Figure 2:**
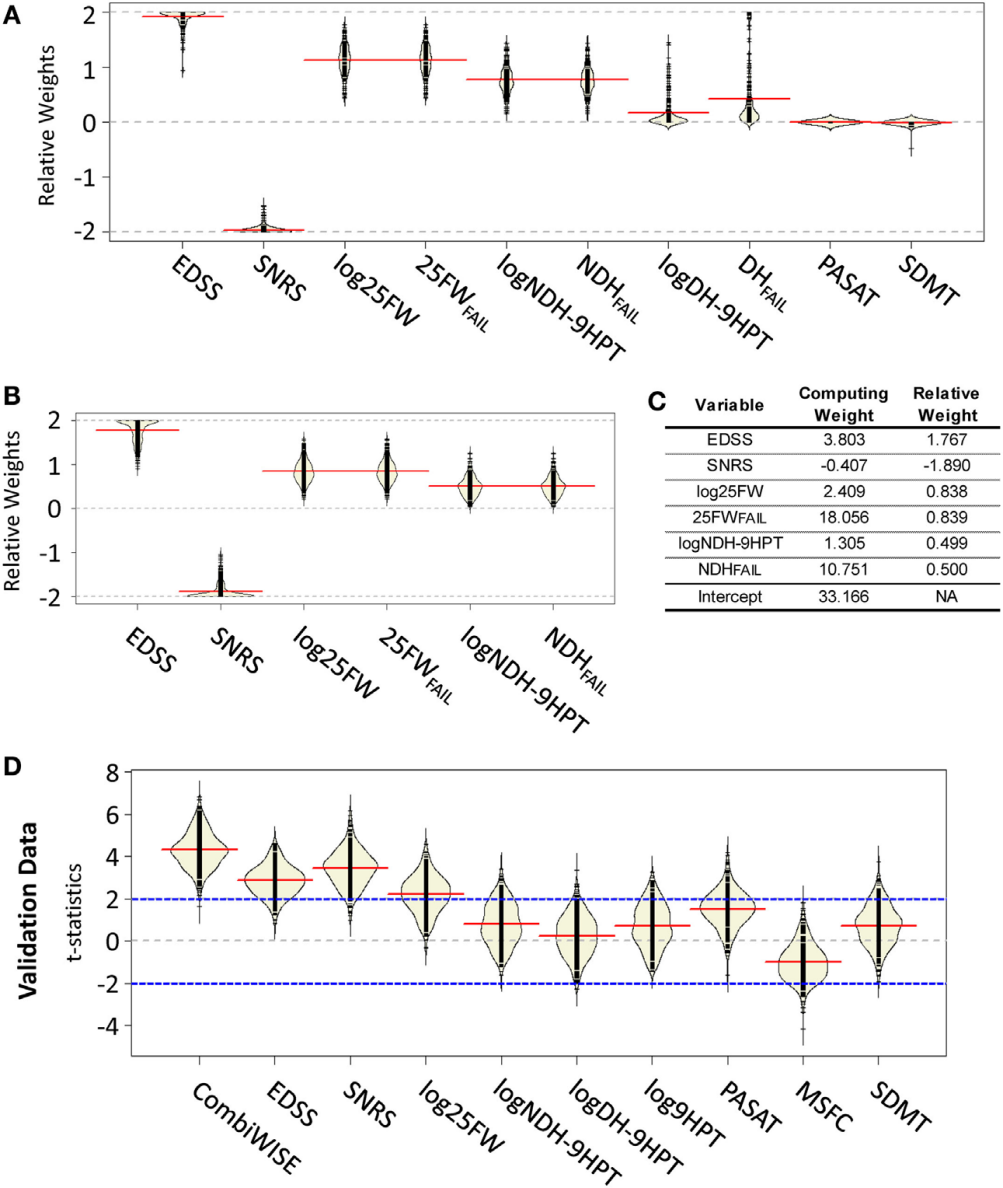
**Development of mathematically optimized CombiWISE scale**. **(A)** A bean plot (25) of variability in relative weights constructed from 200 permutations of training data using the genetic algorithm. **(B)** A bean plot of variability in weights for optimization across 500 permutations of training data using reduced set of clinical scales. **(A,B)** Black lines represent the individual weight results, red lines show the average of weights for each contributing scale, yellow areas represent non-parametric density curves of distribution of individual weights. **(C)** Mean relative weights and computing weights from the contributing clinical scales for CombiWISE calculation. Relative weights allow for comparing the level of contribution of individual scales while the computing weights are rescaled versions that produce a metric ranging from 0 to 100. **(D)** A bean plot of 500 permutations of validation data test-statistics for CombiWISE in addition to individual clinical scales and the Multiple Sclerosis Functional Composite (MSFC) scale. Black lines represent individual test statistics, red lines show average of test statistics for each scale, yellow areas represent non-parametric density curves of individual test statistics, blue lines correspond to cut-offs for 5% significance level tests.

The 200 permutations of the training/validation data for the weights generated from the attempt to use all 10 measured clinical variables [i.e., 9HPT was evaluated independently for the dominant (logDH-9HPT) or non-dominant hand (logNDH-9HPT) and the failure to perform either of them (DH_FAIL_, NDH_FAIL_) or log25FW (25FW_FAIL_) were captured as separate variables, leading to a total of four 9HPT and two 25FW measures tested] demonstrated that cognitive scales (PASAT and SDMT) were always at the boundary of 0, because their direction of change was often opposite to expected clinical progression (i.e., they either did not change or demonstrated a learning effect; Figure 2A). Surprisingly, permutations also revealed differences between logDH-9HPT and logNDH-9HPT, with the latter achieving higher weights, while logDH-9HPT weights were close to zero in most cases. Consequently, to reduce variability of CombiWISE, we removed the aforementioned four clinical outcomes that did not reliably capture disease progression. Five hundred additional GAs (using the same constrained permutation procedure of training/validation data splits) with the remaining six clinical variables (Figure 2B) achieved mean weights that were proportionally comparable to the weights utilized in the conceptually generated CombiWISE v.0 (i.e., weights based on measured *z*-scores in the IPPOMS1 cohort), with following hierarchy: SNRS > EDSS > log25FW = 25FW_FAIL_ > logNDH-9HPT = NDH_FAIL_ (Figure 2C). The measured weights were rescaled so that optimized CombiWISE ranges from 0 to 100 (higher numbers correspond to increasing disability), calculated based on the following formula:

$$\begin{array}{c} \text{CombiWISE}=33.166+3.803*\text{EDSS}-0.407*\text{SNRS}+2.409*\text{log}25\text{FW}+18.056*25{\text{FW}}_{\text{FAIL}}\\ \;+1.305*\text{ logNDH-}9\text{HPT}+10.751*{\text{ NDH}}_{\text{FAIL}} \end{array}$$

In order to assess the performance of the optimized CombiWISE, we compared the resulting test statistics for 500 permutations of the withheld validation data sets for the *t*-statistics for the linear time change in the mixed model (Figure 2D). We observed that CombiWISE outperformed all other clinical scales. In 93.4% of the 500 permuted validation datasets, CombiWISE generates a larger test statistic than SNRS, which is the highest performing single clinical scale, with an average gain of 0.85 *t*-statistic units and a maximum gain of over 2.5 units. CombiWISE also outperformed EDSS and log25FW in over 97% of the permuted validation datasets with average gains of approximately 1.45 and 2 *t*-statistic units, respectively. This gain in *t*-statistic corresponds to higher power in detecting clinical changes over time, particularly if the changes are relatively small (Figure S4 in Supplementary Material), as would be expected in a Phase II trial.

**Figure 3:**
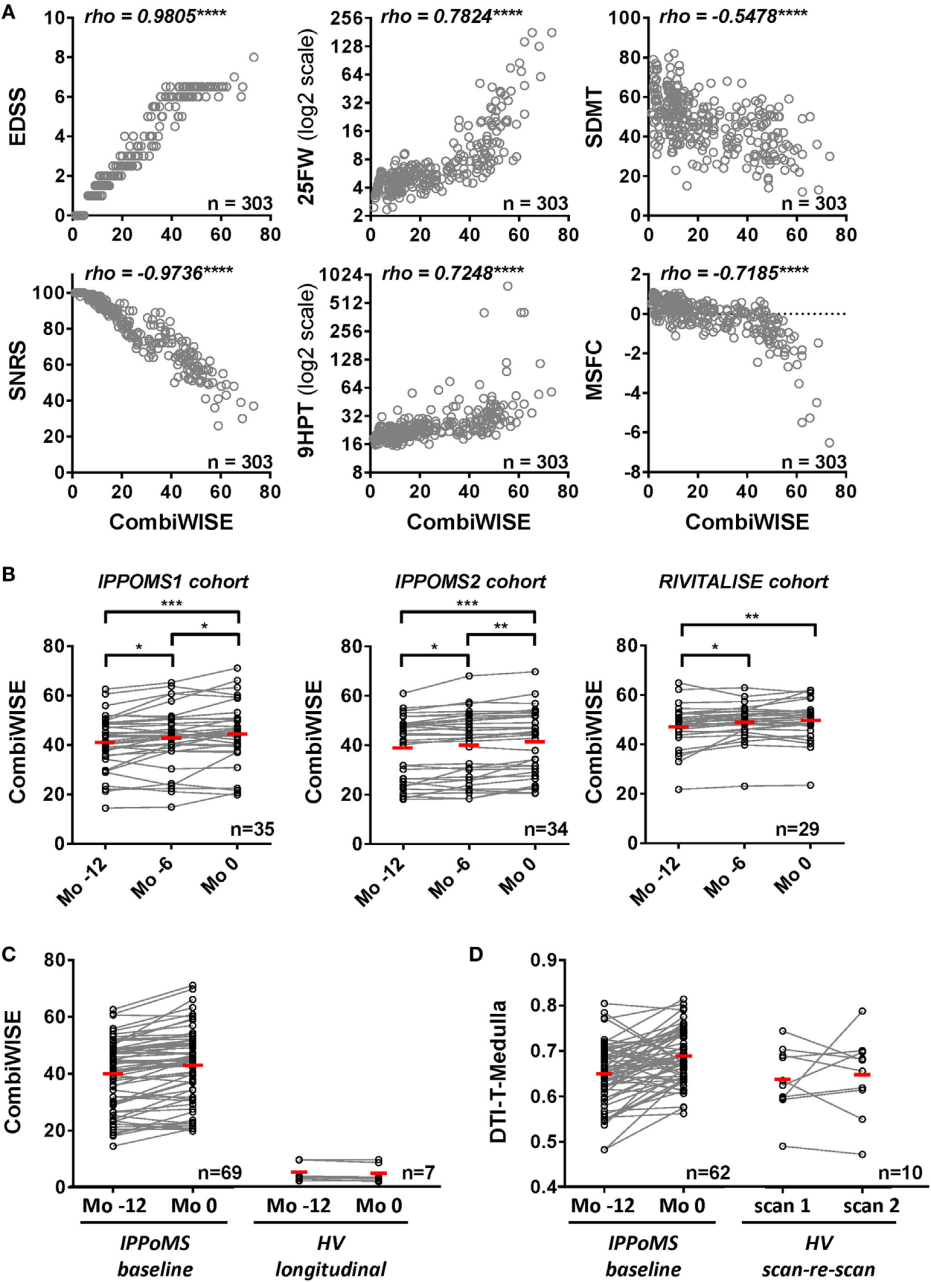
**Combinatorial weight-adjusted disability score (CombiWISE) correlates highly with other clinical scales and shows statistically significant progression in three independent cohorts**. **(A)** Spearman correlations between CombiWISE and standard clinical scores (EDSS, SNRS, 25FW, 9HPT, SDMT, and MSFC) in the cross-sectional cohort of 303 untreated subjects with different types of MS, other inflammatory and non-inflammatory CNS conditions, and healthy volunteers. The *Y*-axis scales for 25FW and 9HTP are log2-transformed; ****p < 0.0001. **(B)** Longitudinal data for CombiWISE calculated from clinical scores collected every 6 months (Mo −12, Mo −6, and Mo 0) during the pre-treatment baseline for PPMS subjects in the IPPoMS trial and for a cohort of 29 untreated SPMS subjects showing statistically significant worsening of the clinical status over periods of 6 to 12 months in both PPMS and SPMS subjects. Statistical significance was determined by one-way ANOVA test on repeated measures. The red bars show mean for each group, *p < 0.05, **p < 0.01, ***p < 0.001, displayed that *p*-values were adjusted for multiple comparisons by Holm–Sidak test. **(C)** Longitudinal data for CombiWISE collected during the pre-treatment baseline of the IPPoMS trial show statistically significant increase in CombiWISE over 1 year. CombiWISE data collected on healthy subjects with a yearly follow-up visit show no overlap with the data of moderately to severely disabled PPMS patients, as well as no appreciable change over 1 year. Red bars represent the mean of the group. **(D)** Comparison of measured change and the overlap of values between IPPoMS and HV cohorts for the best-performing DTI biomarker (axial diffusivity of the medulla). Red bars represent the mean of the group.

CombiWISE correlates strongly with all clinical scales, including cognitive SDMT (which does not contribute to CombiWISE) in the cross-sectional evaluation of >300 untreated neurological patients and HVs (Figure 3A). Furthermore, CombiWISE can reliably detect linear progression of clinical disability in all three progressive MS cohorts, often even in intervals as short as 6 months (Figure 3B). In contrast to MRI measures, which have generally high technical/biological variability (i.e., SNR computed as the average of absolute yearly change in patients divided by the average of absolute scan-re scan difference in HVs; Table S3 in Supplementary Material), with successful MRI biomarkers having SNR between 1.45 and 2.94, CombiWISE has SNR 7.66, even when we used a more stringent definition of SNR, computed as average absolute yearly difference in patients divided by average absolute yearly changes in HVs. Finally, CombiWISE shows no overlap of values between HVs and progressive MS patients (Figure 3C) in contrast to MRI measures. For comparison, we selected the best-performing MRI variable – radial diffusivity of medulla – that shows complete overlap of the values between HVs and moderately to severely disabled progressive MS patients (Figure 3D).

### Power Analysis

**Figure 4:**
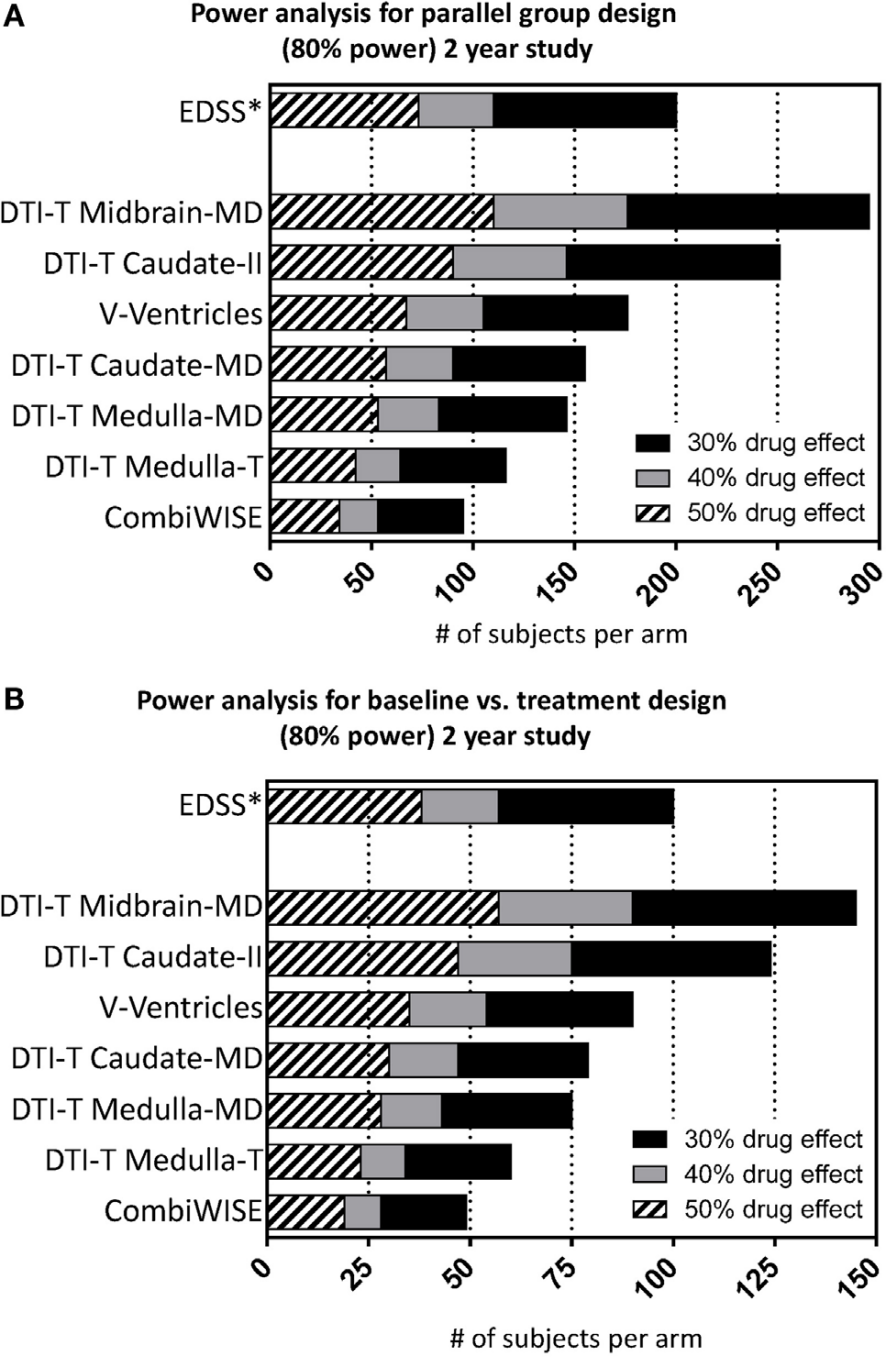
**Power analysis for the selected seven statistically significant variables**. Power analysis shows number of required subjects per arm (accumulated sample size as effect size drops across bars) considering 80% nominal power, statistical significance level of 5% and either **(A)** parallel or **(B)** baseline vs. treatment group design for the 2-year study considering 30% (sum of black, gray, hatched bars), 40% (sum of gray and hatched bars), and 50% (hatched bars) drug effect.

In power calculation, CombiWISE is the best-performing outcome in IPPOMS1 cohort (Figure 4, Table S4 in Supplementary Material). In a parallel-group design, a 2-year treatment study with 1:1 randomization, 34, 53, and 95 subjects per arm are required to detect 50%, 40% versus 30% drug effect, respectively, with 80% power, 5% significance level and two-sided comparisons (Figure 4A). In a baseline-versus-treatment paradigm, 19, 28, and 49 subjects per arm are needed to detect 50%, 40% versus 30% drug effect on CombiWISE, respectively (Figure 4B). We included EDSS in the Figure 4 for direct comparison.

## Discussion

There remains a large unmet need for development of therapies for progressive MS. While at any given time, multiple candidate therapies are available, the present bottleneck resides in the inability to screen them in small, but adequately powered, Phase II trials that can correctly predict efficacy on FDA-accepted clinical endpoint utilized for Phase III trials. This study provides a comprehensive comparison of outcomes in the same patient group(s) within a real-world situation of Phase II clinical trials.

The reason for implementing pre-specified comparison of large number of candidate outcomes into progressive MS trials initiated 7 years ago was the fact that such comparison was lacking in the public domain then, and still is lacking today; surprisingly, the papers that describe candidate new outcomes do not compare these with the traditional outcomes, such as EDSS (2, 10). Nevertheless, the excellent experience with MRI CELs as highly sensitive and reproducible outcome in RRMS poised the MS field to trust the superiority of MRI outcomes over clinical outcomes for progressive MS, in the absence of factual evidence. This belief in superiority of MRI outcomes is virtually universal, as evidenced by the fact that brain atrophy represents the primary outcome in the vast majority of currently ongoing Phase II trials in progressive MS (26).

Despite the fact that we validated six qMRI measures as reliably changing in three progressive MS cohorts over 1 year, we found that they had low to absent correlations with clinical scales and a strong overlap of values between HVs and MS patients. From tested volumetric measures, enlargement of ventricles measured by LesionTOADS was the most reproducible MRI outcome, which outperforms the SIENA brain volume change measurement, but did not outperform CombiWISE in any of the tested cohorts. This MRI outcome showed modest correlations with cognitive scales and the 9HPT, at least in the cross-sectional paradigm, proving its biological relevance. However, its lack of correlation with EDSS (and SNRS, 25FW, or CombiWISE) makes it questionable whether the efficacy on brain atrophy observed in Phase II trials will correctly predict efficacy on clinical outcomes in Phase III studies. In fact, simultaneously measured changes in brain atrophy and clinical parameters were already contradictory in a Phase II progressive MS trial (27). For these multiple reasons, CombiWISE is a better outcome for Phase II trials of progressive MS than ventricular or brain atrophy.

The remaining MRI markers that reproducibly progressed in all three cohorts were DTI biomarkers measured in the head of the caudate nucleus, midbrain, and medulla. These putative biomarkers of CNS tissue integrity were all correlated with each other, but did not correlate with clinical outcomes, even when we re-analyzed data separately for each of the scanners, to avoid influence of the observed “scanner effect” (Figure S6 in Supplementary Material). The most concerning observation was a broad overlap of DTI-derived measurements between HVs who lack neurological disability and moderately-severely disabled MS patients. If the measured yearly increase in DTI outcomes in the MS cohort truly reflected yearly increase in CNS tissue destruction, then extrapolating such a yearly rise in DTI parameters across the long disease duration of our MS cohort would position this cohort way above the HVs. Instead, relatively high scan–rescan variability in HVs in comparison to the yearly changes measured in MS cohorts (i.e., low SNR), prominent scanner effect (Figure S7 in Supplementary Material) and also statistically significant, scan-date-related longitudinal drift of DTI data measured across 3 years (Figure S8 in Supplementary Material), suggest that technical aspects of MRI scanning, rather than biological changes are more likely the cause of the measured yearly increase in DTI parameters.

These observations caution against uncritical interpretations that changes in qMRI parameters reflect unequivocally structural alterations of CNS tissue. An informative review (28) highlights this erroneous assumption: MRI does not measure brain structure; instead, it infers brain structure from the radio-frequency signals of energized hydrogen protons, which are affected by both technical parameters of magnetic fields and magnetic properties of the surrounding tissue. Quantitative data derived from advanced imaging techniques, such as DTI, are computed from mathematical models, parameters of which are influenced by scanner hardware, sequences, and post-processing methods (29). Furthermore, MRI, as a physical–chemical measure, is also influenced by biological phenomena that have nothing to do with the structural integrity of CNS tissue, such as changes in body weight, lipid levels, hydration, and use of alcohol or pharmaceutical agents (28). While these confounding factors are easily controlled in animal experiments from which pathological-MRI correlations have been derived (30, 31), they are impossible to eliminate in the real-world experience of human clinical trials performed on multiple MRI scanners and spanning several years.

Thus, reliance on MRI markers as the primary outcome in progressive MS trials is currently not advisable because of their lack of surrogacy with clinical scales. Surrogacy (32) requires that the biomarker predicts results on clinical outcomes, and does so in a considerably shorter time-period than clinical scales. While correlation with clinical scales is not sufficient, it is nevertheless a prerequisite for surrogacy and none of the MRI markers tested in the current study fulfills this condition in relationship to EDSS, SNRS, 25FW, or CombiWISE. We cannot generalize our conclusions to magnetic resonance spectroscopy or magnetization transfer biomarkers (33–36), which we did not test. Hopefully, future studies of these potentially promising biomarkers will include longitudinal assessment of their variance, influence of scanner(s), and sequences, overlap with data generated in HVs and direct comparison with the simultaneously acquired clinical scales.

Strong to moderate correlations between different clinical scales indicate that these do reflect evolution of underlying disease. Nevertheless, their sensitivity for yearly disease progression is low, as none of them demonstrated statistically significant progression in all three longitudinal cohorts and yearly changes measured by different scales did not correlate. While SDMT correlated stronger with the remaining clinical scales than PASAT did, the modeling permutations found both cognitive tests insensitive to reliably detect yearly progression in small Phase II trials. The idea that a composite score could amplify changes in individual clinical scales has been tested before. Goodkin introduced a composite outcome consisting of designated changes in any of the four utilized clinical scales (37) and demonstrated superior *sensitivity* of such composite (38). An analogous composite primary endpoint was used in recently announced negative trials of natalizumab (ASCEND trial, NCT01416181) and opicinumab (anti-Lingo-1 SYNERGY trial, NCT01864148). Unfortunately, the *specificity* of such an inclusive composite has not been published. The National MS Society Clinical Outcomes Assessment Task Force recommended development of a composite clinical measure in which individual components “should have high *reliability*” (39). The result of this effort was the MSFC (10), introduced without direct comparison to EDSS. In follow-up studies, MSFC change had considerably lower power than EDSS for detecting sustained disability in PPMS subjects (40). Our measurements concur with this conclusion.

In contrast to aforementioned efforts, we used statistical modeling to optimize a composite clinical metric that “weighs” simultaneously captured data from several clinical scales, selected based on their ability to detect MS disease progression in a majority of modeling cohort permutations. CombiWISE is based on the intersection of these scales, benefiting from the noise-limiting feature of combining partially overlapping measurements. Strong correlations of CombiWISE with traditional clinical outcomes observed in multiple cohorts and its excellent SNR fully support the stated conceptual advantages of this scale; because EDSS represents only 28% of the CombiWISE score, retaining strong, statistically significant correlations between changes in CombiWISE and EDSS in a small, year-long study is actually not intuitive (see Figure S5 in Supplementary Material for formal assessment of this statement). The reason why CombiWISE is more than twice as sensitive as EDSS in detecting progression of disability lies in the discreteness of EDSS: while a patient may remain on any given EDSS step for a long time, CombiWISE can detect continuous disease progression as measured by three alternative clinical scales. Yet, thanks to the strong correlation between CombiWISE and EDSS, one can calculate from the resulting regression slopes that 1 point change in EDSS corresponds on average to a 7.50 point change on CombiWISE with a standard error of 0.10, allowing extrapolation of clinical meaning from the CombiWISE measurements. Finally, CombiWISE provides approximately normally distributed data (Figure S9 in Supplementary Material), permitting the use of parametric statistical techniques even in small cohorts. We observed that both conceptually devised and the numerically-optimized version of CombiWISE detect significant disease progression in all three longitudinal progressive MS cohorts, in intervals as short as 6 months. Thus, using CombiWISE as a continuous variable captured every 6 months should provide further advantage over event-driven outcomes, such as the one used in a trial of ocrelizumab in PPMS (ORATORIO trial; NCT01194570). While relatively low numbers of patients in each of the three independent longitudinal cohorts (i.e., *N* = 29–35) may be viewed as a limitation, it proves that CombiWISE reproducibly measures yearly disease progression in cohorts that correspond in size to the treatment versus placebo arms of the economical Phase II trials.

In conclusion, CombiWISE has validated as the most sensitive clinical outcome for progressive MS. It has consistently higher sensitivity for detecting longitudinal changes in progressive MS in comparison to MRI measures of brain atrophy, currently broadly utilized in Phase II progressive MS trials. In contrast to all tested MRI measures, CombiWISE can predict changes in EDSS, presently used for regulatory approval. Substituting EDSS with CombiWISE requires over 100 fewer subjects per arm (200 versus 95) in a parallel-group design to detect a 30% drug effect in a 2-year study.

## Author Contributions

BB designed and supervised the study. PK, IC, GN, TW, CB, MG, and BB analyzed the data and drafted the manuscript and figures. DG, MT, IC, WK, BS, JO, KF, and TL collected or generated data and contributed to reviewing and editing the manuscript.

## Conflict of Interest Statement

The authors declare that the research was conducted in the absence of any commercial or financial relationships that could be construed as a potential conflict of interest.

## Abbreviations

Δ, relative yearly percentage change; 25FW, 25 foot walk; 9HPT, 9 hole peg test; CEL, contrast-enhancing lesion; CIS, clinically isolated syndrome; CMCT, central motor conduction time; CNR, contrast-to-noise ratio; CNS, central nervous system; CombiWISE, combinatorial weight-adjusted disability score; DTI, diffusion tensor imaging; EDSS, expanded disability status scale; FRD, false-rate discovery; HV, healthy volunteer; MRI, magnetic resonance imaging; MS, multiple sclerosis; MSFC, multiple sclerosis functional composite; NIND, non-inflammatory neurological disorders; OCT, optical coherence tomography; OIND, other inflammatory neurological disorders; PASAT, paced auditory serial addition test; PPMS, primary progressive MS; qMRI, quantitative MRI; qT1, quantitative T1; RIS, radiologically isolated syndrome; RNFL, retinal nerve fiber layer; ROI, region of interest; RRMS, relapsing-remitting MS; SC, spinal cord; SDMT, symbol digit modality test; SNR, signal-to-noise ratio; SNRS, Scripps neurological rating scale; SPMS, secondary progressive MS.

## References

[1] Wolinsky JS Narayana PA O’Connor P Coyle PK Ford C Johnson K Glatiramer acetate in primary progressive multiple sclerosis: results of a multinational, multicenter, double-blind, placebo-controlled trial. Ann Neurol (2007) 61(1):14–24. 10.1002/ana.21079 17262850

[2] Altmann DR Jasperse B Barkhof F Beckmann K Filippi M Kappos LD Sample sizes for brain atrophy outcomes in trials for secondary progressive multiple sclerosis. Neurology (2009) 72(7):595–601. 10.1212/01.wnl.0000335765.55346.fc 19005170 PMC2818185

[3] Harrison DM Caffo BS Shiee N Farrell JA Bazin PL Farrell SK Longitudinal changes in diffusion tensor-based quantitative MRI in multiple sclerosis. Neurology (2011) 76(2):179–86. 10.1212/WNL.0b013e318206ca61 21220722 PMC3030233

[4] Frost C Kenward MG Fox NC. Optimizing the design of clinical trials where the outcome is a rate. Can estimating a baseline rate in a run-in period increase efficiency? Stat Med (2008) 27(19):3717–31. 10.1002/sim.3280 18484598

[5] Smith SM Zhang Y Jenkinson M Chen J Matthews PM Federico A Accurate, robust, and automated longitudinal and cross-sectional brain change analysis. Neuroimage (2002) 17(1):479–89. 10.1006/nimg.2002.1040 12482100

[6] Komori M Lin YC Cortese I Blake A Ohayon J Cherup J Insufficient disease inhibition by intrathecal rituximab in progressive multiple sclerosis. Ann Clin Transl Neurol (2016) 3(3):166–79. 10.1002/acn3.293 27042677 PMC4774261

[7] SAS Institute Inc. SAS/STAT^®^9.22 User’s Guide. Cary, NC: SAS Institute (2010).

[8] Kurtzke JF. Rating neurologic impairment in multiple sclerosis: an expanded disability status scale (EDSS). Neurology (1983) 33(11):1444–52. 10.1212/WNL.33.11.1444 6685237

[9] Sipe JC Knobler RL Braheny SL Rice GP Panitch HS Oldstone MB. A neurologic rating scale (NRS) for use in multiple sclerosis. Neurology (1984) 34(10):1368–72. 10.1212/WNL.34.10.1368 6541311

[10] Fischer JS Rudick RA Cutter GR Reingold SC. The multiple sclerosis functional composite measure (MSFC): an integrated approach to MS clinical outcome assessment. National MS society clinical outcomes assessment task force. Mult Scler (1999) 5(4):244–50. 10.1177/135245859900500409 10467383

[11] Hallett M. Transcranial magnetic stimulation: a primer. Neuron (2007) 55(2):187–99. 10.1016/j.neuron.2007.06.026 17640522

[12] Duan Q van Gelderen P Duyn J. Improved Bloch-Siegert based B1 mapping by reducing off-resonance shift. NMR Biomed (2013) 26(9):1070–8. 10.1002/nbm.2920 23355474 PMC3669656

[13] Deoni SC. High-resolution T1 mapping of the brain at 3T with driven equilibrium single pulse observation of T1 with high-speed incorporation of RF field inhomogeneities (DESPOT1-HIFI). J Magn Reson Imaging (2007) 26(4):1106–11. 10.1002/jmri.21130 17896356

[14] Shiee N Bazin PL Ozturk A Reich DS Calabresi PA Pham DL. A topology-preserving approach to the segmentation of brain images with multiple sclerosis lesions. Neuroimage (2010) 49(2):1524–35. 10.1016/j.neuroimage.2009.09.005 19766196 PMC2806481

[15] Chang LC Jones DK Pierpaoli C. RESTORE: robust estimation of tensors by outlier rejection. Magn Reson Med (2005) 53(5):1088–95. 10.1002/mrm.20426 15844157

[16] Landman BA Farrell JA Jones CK Smith SA Prince JL Mori S. Effects of diffusion weighting schemes on the reproducibility of DTI-derived fractional anisotropy, mean diffusivity, and principal eigenvector measurements at 1.5T. Neuroimage (2007) 36(4):1123–38. 10.1016/j.neuroimage.2007.02.056 17532649 PMC12008999

[17] Scrucca L GA: a package for genetic algorithms in R. J Stat Softw (2013) 53(4):1–37. 10.18637/jss.v053.i04

[18] R Development Core Team. R: A Language and Environment for Statistical Computing. Vienna: R Foundation for Statistical Computing (2016). Available from: http://www.r-project.org./

[19] Pinheiro J Bates D Mixed-Effects Models in S and S-PLUS. New York, NY: Springer-Verlag (2000).

[20] Pinheiro J Bates D DebRoy S Sarkar D R Core Team nlme: Linear and Non-linear Mixed Effects Models. R package Version 3.1-117. (2014). Available from: http://CRAN.R-project.org/package=nlme

[21] Bock M Brandt AU Kuchenbecker J Dorr J Pfueller CF Weinges-Evers N Impairment of contrast visual acuity as a functional correlate of retinal nerve fibre layer thinning and total macular volume reduction in multiple sclerosis. Br J Ophthalmol (2012) 96(1):62–7. 10.1136/bjo.2010.193581 21378002

[22] Holland J Adaptation in Natural and Artificial Systems. Ann Arbor: The University of Michigan Press (1975).

[23] Goldberg D Genetic Algorithms in Search, Optimization, and Machine Learning. Boston: Addison-Wesley (1989).

[24] Sivanandam S Deepa S Introduction to Genetic Algorithms. Berlin: Springer-Verlag (2008).

[25] Kampstra P Beanplot: a boxplot alternative for visual comparison of distributions. J Stat Softw (2008) 28(1):1–9. 10.18637/jss.v028.c01

[26] Ontaneda D Fox RJ Chataway J. Clinical trials in progressive multiple sclerosis: lessons learned and future perspectives. Lancet Neurol (2015) 14(2):208–23. 10.1016/S1474-4422(14)70264-9 25772899 PMC4361791

[27] Kapoor R Furby J Hayton T Smith KJ Altmann DR Brenner R Lamotrigine for neuroprotection in secondary progressive multiple sclerosis: a randomised, double-blind, placebo-controlled, parallel-group trial. Lancet Neurol (2010) 9(7):681–8. 10.1016/S1474-4422(10)70131-9 20621711

[28] Weinberger DR Radulescu E. Finding the elusive psychiatric “lesion” with 21st-century neuroanatomy: a note of caution. Am J Psychiatry (2016) 173(1):27–33. 10.1176/appi.ajp.2015.15060753 26315983

[29] Jones DK Cercignani M. Twenty-five pitfalls in the analysis of diffusion MRI data. NMR Biomed (2010) 23(7):803–20. 10.1002/nbm.1543 20886566

[30] Zhang J Jones MV McMahon MT Mori S Calabresi PA. *In vivo* and *ex vivo* diffusion tensor imaging of cuprizone-induced demyelination in the mouse corpus callosum. Magn Reson Med (2012) 67(3):750–9. 10.1002/mrm.23032 21656567 PMC3170659

[31] Jones DK Knosche TR Turner R. White matter integrity, fiber count, and other fallacies: the do’s and don’ts of diffusion MRI. Neuroimage (2013) 73:239–54. 10.1016/j.neuroimage.2012.06.081 22846632

[32] Prentice RL. Surrogate endpoints in clinical trials: definition and operational criteria. Stat Med (1989) 8(4):431–40. 10.1002/sim.4780080407 2727467

[33] Arnold DL. Evidence for neuroprotection and remyelination using imaging techniques. Neurology (2007) 68(22 Suppl 3):S83–90. 10.1212/01.wnl.0000275237.28259.9d 17548574

[34] Khaleeli Z Sastre-Garriga J Ciccarelli O Miller DH Thompson AJ. Magnetisation transfer ratio in the normal appearing white matter predicts progression of disability over 1 year in early primary progressive multiple sclerosis. J Neurol Neurosurg Psychiatry (2007) 78(10):1076–82. 10.1136/jnnp.2006.107565 17287235 PMC2117577

[35] Khaleeli Z Altmann DR Cercignani M Ciccarelli O Miller DH Thompson AJ. Magnetization transfer ratio in gray matter: a potential surrogate marker for progression in early primary progressive multiple sclerosis. Arch Neurol (2008) 65(11):1454–9. 10.1001/archneur.65.11.1454 19001163

[36] Llufriu S Kornak J Ratiney H Oh J Brenneman D Cree BA Magnetic resonance spectroscopy markers of disease progression in multiple sclerosis. JAMA Neurol (2014) 71(7):840–7. 10.1001/jamaneurol.2014.895 24839987

[37] Goodkin DE Rudick RA VanderBrug Medendorp S Greene T Schwetz KM Fischer J Low-dose (7.5 mg) oral methotrexate for chronic progressive multiple sclerosis. Design of a randomized, placebo-controlled trial with sample size benefits from a composite outcome variable including preliminary data on toxicity. Online J Curr Clin Trials (1992) 19:7723. 1343611

[38] Goodkin DE Rudick RA VanderBrug Medendorp S Daughtry MM Schwetz KM Fischer J Low-dose (7.5 mg) oral methotrexate reduces the rate of progression in chronic progressive multiple sclerosis. Ann Neurol (1995) 37(1):30–40. 10.1002/ana.410370108 7818255

[39] Rudick R Antel J Confavreux C Cutter G Ellison G Fischer J Recommendations from the national multiple sclerosis society clinical outcomes assessment task force. Ann Neurol (1997) 42(3):379–82. 10.1002/ana.410420318 9307263

[40] Kragt JJ Thompson AJ Montalban X Tintore M Rio J Polman CH Responsiveness and predictive value of EDSS and MSFC in primary progressive MS. Neurology (2008) 70(13 Pt 2):1084–91. 10.1212/01.wnl.0000288179.86056.e1 18184917

