# Supplementary material for: Development of a Sensitive Outcome for Economical Drug Screening for Progressive Multiple Sclerosis Treatment
Source: Front Neurol. 2016 Aug 15;7:131. doi: 10.3389/fneur.2016.00131 (PMC4983704; doi:10.3389/fneur.2016.00131)
Supplement: Supplementary file 1 [file presentation_1.pdf]

Supplementary Material

Development of a sensitive outcome for economical drug screening for progressive multiple sclerosis treatment

Peter Kosa, Danish Ghazali, Makoto Tanigawa, Chris Barbour, Irene Cortese, William Kelley, Blake Snyder, Joan Ohayon, Kaylan Fenton, Tanya Lehky, Tianxia Wu, Mark Greenwood, Govind Nair and Bibiana Bielekova*

*** Correspondence:** Bibi.Bielekova@nih.gov

Figure S1

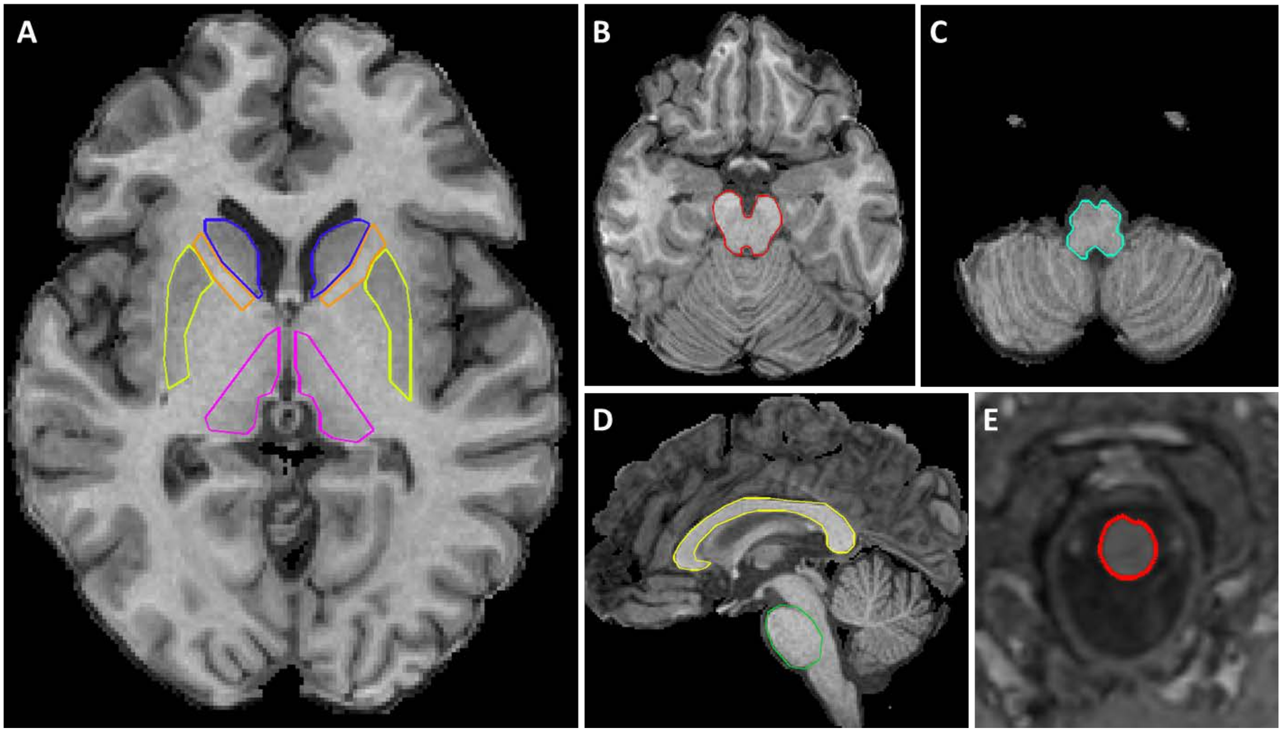

Figure S1: Examples of regions of interest drawn for volume, qT1, and DTIJ measurements.

Regions of interest drawn on various slices in the brain: **(A)** caudate in blue, putamen in yellow, internal capsule in orange, thalamus in magenta, **(B)** midbrain in red, **(C)** medulla in cyan, **(D)** corpus callosum in yellow, pons in green, and spinal cord at the level of **(E)** dens on a representative subject.

Figure S2

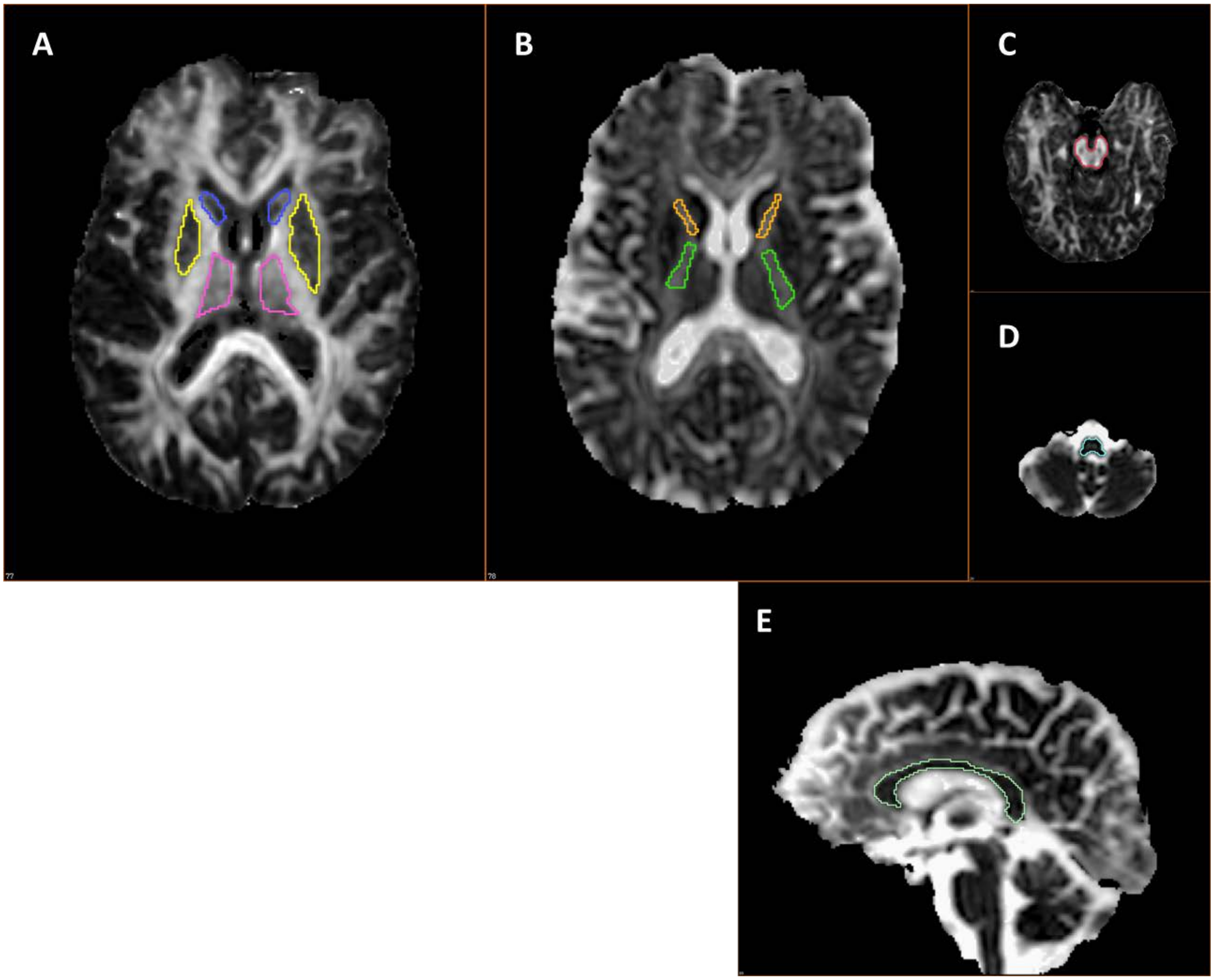

Figure S2: Examples of regions of interest drawn for DTI measurements.

Regions of interest drawn on various slices in the brain: **(A)** caudate in blue, putamen in yellow, thalamus in magenta, drawn on a fractional anisotropy image, **(B)** anterior internal capsule in orange, posterior internal capsule in green, overlaid on parallel diffusivity image, **(C)** midbrain in red, overlaid on a fractional anisotropy image, **(D)** medulla in cyan and **(E)** corpus callosum in green, both overlaid on mean diffusivity images.

Figure S3

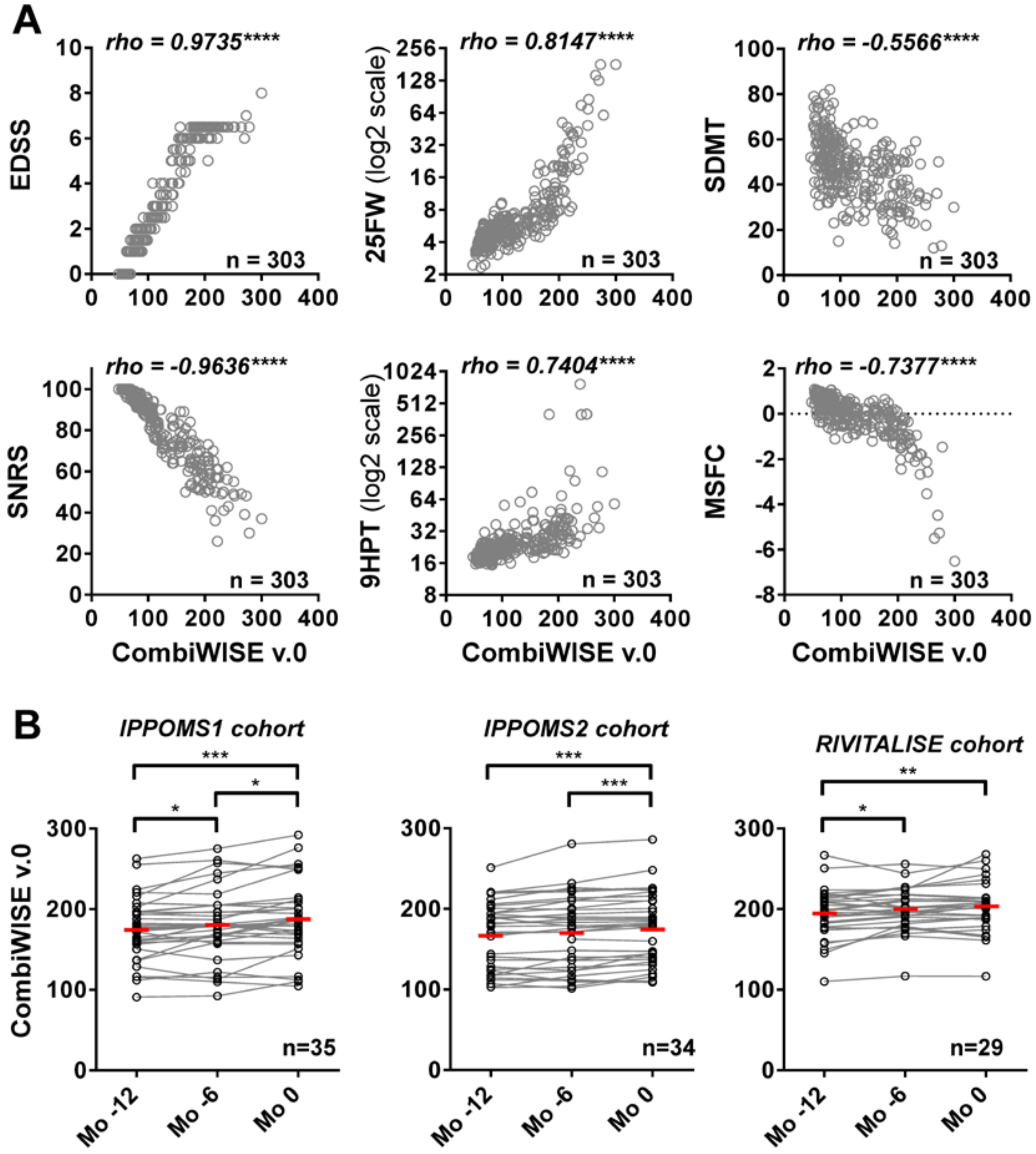

Figure S3: Conceptual model of Combinatorial weight-adjusted disability score (CombiWISE v.0) correlates highly with standard clinical scales and shows significant longitudinal progression in untreated PPMS and SPMS patients

(A) Spearman correlations between CombiWISE v.0 and standard clinical scores (EDSS, SNRS, 25FW, 9HPT, SDMT, and MSFC) in the cross-sectional cohort of 303 untreated subjects with different types of MS, other inflammatory and non-inflammatory CNS conditions, and healthy volunteers. The Y- axis scales for 25FW and 9HTP are log2-transformed; **** p<0·0001 (B) Longitudinal data for CombiWISE calculated from clinical score collected every six months (Mo -12, Mo -6, Mo 0) during the pre-treatment baseline for PPMS subjects in the IPPOMS1 and IPPOMS2 cohorts and for SPMS subjects in the RIVITALISE cohort showing statistically significant worsening

of the clinical status over periods of 6 to 12 months in all three cohorts. Statistical significance was determined by one-way ANOVA test on repeated measures. The red bars show mean for each group, * $p<0.05$, ** $p<0.01$, *** $p<0.001$, displayed p-values were adjusted for multiple comparisons by Holm-Sidak test.

Conceptual development of CombiWISE v.0:

We considered a combinatorial score, where clinical scales with partially overlapping elements are combined into a score, and the relative importance of contributing clinical scales is proportionally adjusted (weighted) based on z-scores measured in the IPPMS1 cohort. We initially combined SNRS, EDSS and 25FW, the three clinical scales that demonstrated significant progression in IPPMS1 one-year longitudinal study, with relative weights of 0.458, 0.521 and 0.334, respectively (which can be normalized to the weight of EDSS as 1.138 [SNRS], 1 [EDSS] and 0.730 [25FW]). In order to assure that higher numbers signify higher functional deficit, we multiplied EDSS by 10 (to achieve a range of values from 0 [best] to 100 [worst]) and multiplied inverted SNRS by 1.138 (to achieve a range of values from 0 [best] to 113.8 [worst]). Before adding 25FW, we tested whether increasing its linearity (as we observed significant skewness towards high values) by $\log_2$-transformation will improve its z-score. Indeed, we observed increase in z-score from 0.334 to 0.497, while retaining statistical significance. We then multiplied $\log_2$-transformed 25FW values by 14.48 to achieve the highest possible value of 108 (for a maximum of 180 seconds allowed for the test for subjects who can no longer walk), which corresponds proportionally to the new z-score of 0.497. The linear combination of these three modified clinical scales achieved thus far the highest z-score (0.700). However, we tested additional rational modification: the problem with the 25FW in small trials of moderately to severely disabled patients is its ceiling effect; patients who reach non-ambulatory status can no longer progress on 25FW and are also unlikely to experience further progression on EDSS in short-term trials[1]. While SNRS can still capture progression in other clinical domains for these patients, we desired to include another partially overlapping scale (i.e. 9HPT) with the rationale of amplifying true disability while filtering out measurement noise (i.e. previously mentioned repeated measure paradigm). Due to similar skewness towards higher values as observed for 25FW, we $\log_2$-transformed the 9HPT data separately for each hand as well as assigned more appropriate numeric codes for inability to complete 9HPT in less than 5 minutes (i.e. instead of using 777, which is recommended by MSFC scoring manual, we used 300 seconds, which corresponds exactly to the 5 minutes limit for the test). Finally, we multiplied the sum of $\log_2$-transformed 9HPT scores for each hand by 3.49 to achieve the highest possible value of 57 (which corresponds to the observed z-score for transformed 9HPT of 0.263), before adding this score to the remaining three modified components. We also tested addition of cognitive scales, using the same methodology, but observed worsening of the performance of resultant combinatorial scale.

These concepts guided the origin of a combinatorial weight-adjusted disability score (CombiWISE v.0), which is calculated according to the formula:

$$CombiWISE\ v.0 = EDSS*10 + (100 - Scripps\text{-}NRS)*1.138 + (\log_2 25FW)*14.480 + (\log_2 (9HPT\text{-}DH) + \log_2 (9HPT\text{-}NDH))*3.490$$

[1] Kragt JJ, Thompson AJ, Montalban X, Tintore M, Rio J, Polman CH, et al. Responsiveness and predictive value of EDSS and MSFC in primary prossive MS. Neurology. 2008;70(13 Pt 2):1084-91.

Figure S4

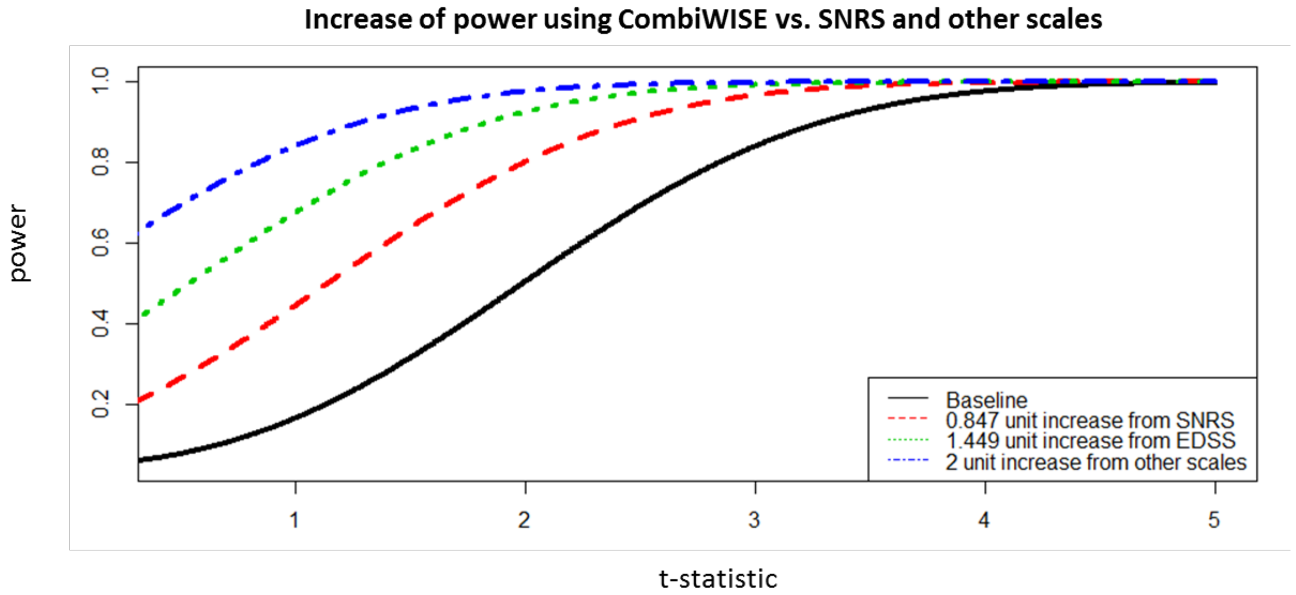

Figure S4: Increase of power in detection of a significant longitudinal change for CombiWISE compared to other standard clinical scales

Power curves for a t-distribution with 70 degrees of freedom for no increase (black line), a 0.85 unit increase (red dashed line), 1.45 unit increase (green dotted line), and 2 unit increase (blue dash-dotted line) corresponding to the average increase in t-statistics from using CombiWISE versus SNRS, EDSS, and other clinical scales, respectively.

Figure S5

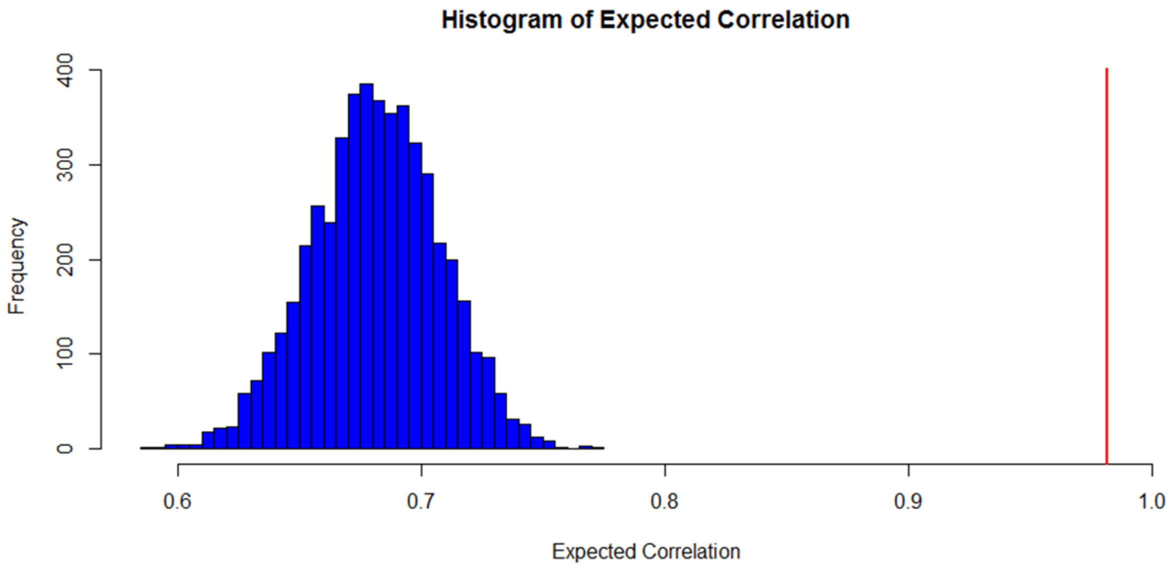

Figure S5: Distribution of expected correlation between EDSS and CombiWISE resulting from EDSS being used in CombiWISE in the N=303 cross-sectional dataset. The red line indicates the observed correlation between EDSS and CombiWISE. We see that the observed correlation always fell above the distribution of expected correlation.

We show that the strong correlation between EDSS and CombiWISE is due to the correlation between EDSS and the other clinical scales used to calculate CombiWISE and not exclusively from EDSS being used in calculating CombiWISE. To demonstrate the strength of the correlation between EDSS and CombiWISE, we calculated a distribution of the expected correlation between CombiWISE and EDSS from EDSS being used in the metric. To generate this, we shuffle the values of EDSS in the cross-sectional dataset and re-compute the value of CombiWISE. This has the effect of de-correlating the values of EDSS with the other clinical scales used to create the metric. Therefore, the correlation between the shuffled EDSS and the re-computed CombiWISE represents the expected correlation between CombiWISE and EDSS from EDSS being used in CombiWISE. This process was repeated 10,000 times to generate the empirical distribution of this expected correlation. We see that our observed correlation is much greater than what we would expect exclusively from EDSS being used in the metric, indicating that the correlation that we observed between EDSS and CombiWISE is stronger than it would be only from EDSS being included in the model. This validates our premise that the strong correlations measured are due to overlapping features of the four contributing clinical scales, the relationship that random simulations broke. The non-intuitive nature of the strong measured correlations between EDSS and CombiWISE is further reinforced by our observations that MSFC, which is also a composite score, has much lower correlations with its three components (i.e., 25FW, 9HPT and PASAT; Figure 1) in the N=98 patient cohort. In contrast, while EDSS, SNRS and 25FW are not correlated in the longitudinal paradigm, CombiWISE correlates strongly with all three measures, confirming that CombiWISE captures those overlapping aspects of contributing clinical scales that reflect sustained disability, while limiting noise.

Figure S6

A

month -12<br>3TA scanner	EDSS	SNRS	25FW	9HPT Avg	PASAT	MSFC	SDMT	CombiWISE	V-Ventricles	DTI-II-Caudate	DTI-T-Medulla	DTI-MD-Caudate	DTI-MD-Medulla	DTI-MD-Midbrain
EDSS		-0.736<br><0.00001	0.692<br>0.00002	0.482<br>0.00703	-0.224<br>0.24304	-0.606<br>0.00049	-0.353<br>0.07111	0.886<br><0.00001	0.304<br>0.10861	-0.256<br>0.27507	0.248<br>0.29097	-0.175<br>0.46052	0.114<br>0.63311	0.190<br>0.42354
SNRS	-0.736<br><0.00001		-0.618<br>0.00027	-0.418<br>0.02168	0.469<br>0.01022	0.625<br>0.00029	0.467<br>0.01412	-0.880<br><0.00001	-0.146<br>0.45121	0.086<br>0.71814	-0.097<br>0.68280	0.005<br>0.98235	-0.089<br>0.70866	-0.091<br>0.70157
25FW	0.692<br>0.00002	-0.618<br>0.00027		0.403<br>0.02708	-0.229<br>0.23266	-0.627<br>0.00027	-0.429<br>0.02539	0.828<br><0.00001	0.226<br>0.23928	-0.329<br>0.15623	0.078<br>0.74315	-0.395<br>0.08435	0.008<br>0.97490	-0.114<br>0.63139
9HPT	0.482<br>0.00703	-0.418<br>0.02168	0.403<br>0.02708		-0.313<br>0.09875	-0.830<br><0.00001	-0.843<br><0.00001	0.570<br>0.00102	0.420<br>0.02313	-0.512<br>0.02094	-0.058<br>0.80836	-0.270<br>0.24957	-0.071<br>0.76707	-0.056<br>0.81571
PASAT	-0.224<br>0.24304	0.469<br>0.01022	-0.229<br>0.23266	-0.313<br>0.09875		0.610<br>0.00044	0.334<br>0.08853	-0.395<br>0.03377	-0.294<br>0.12939	-0.274<br>0.25601	-0.515<br>0.02406	-0.494<br>0.03163	-0.432<br>0.06451	-0.490<br>0.03306
MSFC	-0.606<br>0.00049	0.625<br>0.00029	-0.627<br>0.00027	-0.830<br><0.00001	0.610<br>0.00044		0.747<br><0.00001	-0.774<br><0.00001	-0.445<br>0.01765	0.335<br>0.16080	-0.323<br>0.17767	0.189<br>0.43722	-0.270<br>0.26328	-0.046<br>0.85290
SDMT	-0.353<br>0.07111	0.467<br>0.01412	-0.429<br>0.02539	-0.843<br><0.00001	0.334<br>0.08853	0.747<br>0.00001		-0.567<br>0.00203	-0.444<br>0.02315	0.416<br>0.08584	0.145<br>0.56612	0.207<br>0.40976	0.201<br>0.42425	0.204<br>0.41697
CombiWISE	0.886<br><0.00001	-0.880<br><0.00001	0.828<br><0.00001	0.570<br>0.00102	-0.395<br>0.03377	-0.774<br><0.00001	-0.567<br>0.00203		0.271<br>0.15514	-0.268<br>0.25389	0.177<br>0.45420	-0.209<br>0.37647	0.141<br>0.55221	0.054<br>0.82067
V-Ventricles	-0.256<br>0.10861	0.086<br>0.45121	-0.329<br>0.23928	-0.512<br>0.02313	-0.274<br>0.12939	0.335<br>0.01765	0.416<br>0.02315	-0.268<br>0.15514	-0.167	0.48182	0.235<br>0.56510	0.707<br>0.92480	0.253<br>0.62238	0.469<br>0.89984
DTI-II-Caudate	0.248<br>0.27507	-0.097<br>0.71814	0.078<br>0.15623	-0.058<br>0.02094	-0.515<br>0.25601	-0.323<br>0.16080	0.145<br>0.08584	0.177<br>0.25389	0.137<br>0.48182	0.235	0.31948	0.238<br>0.00049	0.901<br>0.28255	0.056<br>0.03690
DTI-T-Medulla	-0.215<br>0.29097	0.013<br>0.68280	-0.405<br>0.74315	-0.270<br>0.80836	-0.494<br>0.02406	0.212<br>0.17767	0.207<br>0.56612	-0.188<br>0.45420	0.023<br>0.56510	0.707<br>0.31948		0.238<br>0.31313	0.292<br><0.00001	0.553<br>0.81577
DTI-MD-Caudate	-0.175<br>0.46052	0.005<br>0.98235	-0.395<br>0.08435	-0.270<br>0.24957	-0.494<br>0.03163	0.189<br>0.43722	0.207<br>0.40976	-0.209<br>0.37647	0.023<br>0.92480	0.707<br>0.00049	0.238<br>0.31313		0.292<br>0.21203	0.553<br>0.01137
DTI-MD-Medulla	0.114<br>0.63311	-0.089<br>0.70866	0.008<br>0.97490	-0.071<br>0.76707	-0.432<br>0.06451	-0.270<br>0.26328	0.201<br>0.42425	0.141<br>0.55221	0.117<br>0.62238	0.253<br>0.28255	0.901<br><0.00001	0.292<br>0.21203		0.027<br>0.90981
DTI-MD-Midbrain	0.190<br>0.42354	-0.091<br>0.70157	-0.114<br>0.63139	-0.056<br>0.81571	-0.490<br>0.03306	-0.046<br>0.85290	0.204<br>0.41697	0.054<br>0.82067	-0.030<br>0.89984	0.469<br>0.03690	0.056<br>0.81577	0.553<br>0.01137	0.027<br>0.90981	

B

month -12<br>3TD scanner	EDSS	SNRS	25FW	9HPT Avg	PASAT	MSFC	SDMT	CombiWISE	V-Ventricles	DTI-II-Caudate	DTI-T-Medulla	DTI-MD-Caudate	DTI-MD-Medulla	DTI-MD-Midbrain
EDSS		-0.702<br><0.00001	0.862<br><0.00001	0.562<br><0.00001	-0.197<br>0.11528	-0.681<br><0.00001	-0.328<br>0.00677	0.913<br><0.00001	0.100<br>0.42311	0.083<br>0.52206	0.114<br>0.37583	0.033<br>0.80104	0.041<br>0.74922	-0.045<br>0.72728
SNRS	-0.702<br><0.00001		-0.579<br><0.00001	-0.478<br>0.00004	0.073<br>0.56212	0.520<br><0.00001	0.326<br>0.00704	-0.865<br><0.00001	-0.001<br>0.99177	-0.048<br>0.71245	-0.015<br>0.91023	-0.032<br>0.80300	-0.037<br>0.77544	-0.029<br>0.82489
25FW	0.862<br><0.00001	-0.579<br><0.00001		0.546<br><0.00001	-0.181<br>0.14939	-0.717<br><0.00001	-0.286<br>0.01910	0.851<br><0.00001	0.080<br>0.52130	0.059<br>0.64955	0.028<br>0.83136	0.015<br>0.90705	-0.021<br>0.86837	0.010<br>0.93559
9HPT	0.562<br><0.00001	-0.478<br>0.00004	0.546<br><0.00001		-0.196<br>0.11858	-0.819<br><0.00001	-0.426<br>0.00032	0.638<br><0.00001	0.261<br>0.03574	-0.019<br>0.88677	-0.010<br>0.93665	-0.026<br>0.84255	-0.035<br>0.78757	-0.200<br>0.12134
PASAT	-0.197<br>0.11528	0.073<br>0.56212	-0.181<br>0.14939	-0.196<br>0.11858		0.511<br>0.00001	0.629<br><0.00001	-0.150<br>0.23219	-0.367<br>0.00313	-0.064<br>0.63138	-0.197<br>0.13449	-0.047<br>0.72420	-0.228<br>0.08242	-0.109<br>0.41026
MSFC	-0.681<br><0.00001	0.520<br><0.00001	-0.717<br><0.00001	-0.819<br><0.00001	0.511<br>0.00001		0.536<br><0.00001	-0.723<br><0.00001	-0.313<br>0.01263	-0.009<br>0.94746	-0.086<br>0.51706	0.001<br>0.99124	-0.038<br>0.77312	0.041<br>0.75533
SDMT	-0.328<br>0.00677	0.326<br>0.00704	-0.286<br>0.01910	-0.426<br>0.00032	0.629<br><0.00001	0.536<br><0.00001		-0.333<br>0.00582	-0.471<br>0.00008	0.017<br>0.89484	-0.072<br>0.58145	0.065<br>0.61960	-0.083<br>0.52580	0.007<br>0.95964
CombiWISE	0.913<br><0.00001	-0.865<br><0.00001	0.851<br><0.00001	0.638<br><0.00001	-0.150<br>0.23219	-0.723<br><0.00001	-0.333<br>0.00582		0.041<br>0.74523	0.061<br>0.64210	0.061<br>0.63890	0.041<br>0.75229	0.050<br>0.70331	-0.003<br>0.98386
V-Ventricles	0.100<br>0.42311	-0.001<br>0.99177	0.080<br>0.52130	0.261<br>0.03574	-0.367<br>0.00313	-0.313<br>0.01263	-0.471<br>0.00008	0.041<br>0.74523		-0.042<br>0.74834	-0.189<br>0.14872	-0.118<br>0.36886	-0.147<br>0.26388	-0.139<br>0.28938
DTI-II-Caudate	0.083<br>0.52206	-0.048<br>0.71245	0.059<br>0.64955	-0.019<br>0.88677	-0.064<br>0.63138	-0.009<br>0.94746	0.017<br>0.89484	0.061<br>0.64210	-0.042<br>0.74834		0.113<br>0.38297	0.954<br><0.00001	0.148<br>0.25067	0.478<br>0.00009
DTI-T-Medulla	0.114<br>0.37583	-0.015<br>0.91023	0.028<br>0.83136	-0.010<br>0.93665	-0.197<br>0.13449	-0.086<br>0.51706	-0.072<br>0.58145	0.061<br>0.63890	-0.189<br>0.14872	0.113<br>0.38297		0.182<br>0.15734	0.890<br><0.00001	0.335<br>0.00770
DTI-MD-Caudate	0.033<br>0.80104	-0.032<br>0.80300	0.015<br>0.90705	-0.026<br>0.84255	-0.047<br>0.72420	0.001<br>0.99124	0.065<br>0.61960	0.041<br>0.75229	-0.118<br>0.36886	0.954<br><0.00001	0.182<br>0.15734		0.225<br>0.07892	0.541<br><0.00001
DTI-MD-Medulla	0.041<br>0.74922	-0.037<br>0.77544	-0.021<br>0.86837	-0.035<br>0.78757	-0.228<br>0.08242	-0.038<br>0.77312	-0.083<br>0.52580	0.050<br>0.70331	-0.147<br>0.26388	0.148<br>0.25067	0.890<br><0.00001	0.225<br>0.07892		0.363<br>0.00369
DTI-MD-Midbrain	-0.045<br>0.72728	-0.029<br>0.82489	0.010<br>0.93559	-0.200<br>0.12134	-0.109<br>0.41026	0.041<br>0.75533	0.007<br>0.95964	-0.003<br>0.98386	-0.139<br>0.28938	0.478<br>0.00009	0.335<br>0.00770	0.541<br><0.00001	0.363<br>0.00369	

C

longitudinal Δ 3TA scanner	EDSS	SNRS	25FW	9HPT Avg	PASAT	MSFC	SDMT	CombiWISE	V-Ventricles	DTI-II-Caudate	DTI-T-Medulla	DTI-MD-Caudate	DTI-MD-Medulla	DTI-MD-Midbrain
**EDSS**		0.035<br>0.85369	-0.056<br>0.76756	-0.331<br>0.07414	-0.289<br>0.12825	-0.015<br>0.93857	0.115<br>0.57453	**0.507**<br>0.00422	-0.058<br>0.76940	-0.132<br>0.57880	-0.169<br>0.47592	-0.124<br>0.60236	-0.333<br>0.15080	0.056<br>0.81336
**SNRS**	0.035<br>0.85369		-0.206<br>0.27414	-0.243<br>0.19643	0.349<br>0.06353	-0.066<br>0.73399	0.274<br>0.17574	**-0.654**<br>**0.00009**	0.004<br>0.98452	-0.499<br>0.02521	0.014<br>0.95224	-0.411<br>0.07160	-0.044<br>0.85240	-0.159<br>0.50328
**25FW**	-0.056<br>0.76756	-0.206<br>0.27414		0.258<br>0.16893	-0.091<br>0.64002	0.266<br>0.16308	-0.326<br>0.10419	0.450<br>0.01258	0.140<br>0.47698	-0.012<br>0.95985	-0.202<br>0.39426	-0.044<br>0.85515	-0.174<br>0.46201	0.319<br>0.17070
**9HPT**	-0.331<br>0.07414	-0.243<br>0.19643	0.258<br>0.16893		-0.105<br>0.58887	0.102<br>0.59687	**-0.727**<br>**0.00003**	0.119<br>0.53258	-0.056<br>0.77781	0.233<br>0.32268	0.235<br>0.31948	0.236<br>0.31629	0.398<br>0.08181	0.241<br>0.30686
**PASAT**	-0.289<br>0.12825	0.349<br>0.06353	-0.091<br>0.64002	-0.105<br>0.58887		-0.114<br>0.55751	0.228<br>0.26229	-0.329<br>0.08105	-0.090<br>0.65396	-0.239<br>0.32525	-0.291<br>0.22640	-0.205<br>0.39922	-0.279<br>0.24748	-0.107<br>0.66279
**MSFC**	-0.015<br>0.93857	-0.066<br>0.73399	0.266<br>0.16308	0.102<br>0.59687	-0.114<br>0.55751		-0.100<br>0.62741	0.052<br>0.78988	0.184<br>0.35887	0.111<br>0.65239	0.125<br>0.61140	0.374<br>0.11503	0.114<br>0.64204	0.177<br>0.46802
**SDMT**	0.115<br>0.57453	0.274<br>0.17574	-0.326<br>0.10419	**-0.727**<br>**0.00003**	0.228<br>0.26229	-0.100<br>0.62741		-0.291<br>0.14867	0.070<br>0.74081	-0.091<br>0.72969	0.093<br>0.72259	-0.074<br>0.78016	-0.054<br>0.83888	0.118<br>0.65265
**CombiWISE**	**0.507**<br>0.00422	**-0.654**<br>**0.00009**	0.450<br>0.01258	0.119<br>0.53258	-0.329<br>0.08105	0.052<br>0.78988	-0.291<br>0.14867		-0.039<br>0.84218	0.229<br>0.33240	-0.149<br>0.53104	0.161<br>0.49797	-0.141<br>0.55221	0.227<br>0.33567
**V-Ventricles**	-0.058<br>0.76940	0.004<br>0.98452	0.140<br>0.47698	-0.056<br>0.77781	-0.090<br>0.65396	0.184<br>0.35887	0.070<br>0.74081	-0.039<br>0.84218		-0.149<br>0.54232	0.275<br>0.25372	0.025<br>0.92050	0.167<br>0.49527	0.282<br>0.24133
**DTI-II-Caudate**	-0.132<br>0.57880	-0.499<br>0.02521	-0.012<br>0.95985	0.233<br>0.32268	-0.239<br>0.32525	0.111<br>0.65239	-0.091<br>0.72969	0.229<br>0.33240	-0.149<br>0.54232		0.392<br>0.08696	**0.800**<br>**0.00002**	0.412<br>0.07105	0.388<br>0.09097
**DTI-T-Medulla**	-0.169<br>0.47592	0.014<br>0.95224	-0.202<br>0.39426	0.235<br>0.31948	-0.291<br>0.22640	0.125<br>0.61140	0.093<br>0.72259	-0.149<br>0.53104	0.275<br>0.25372	0.392<br>0.08696		**0.502**<br>0.02402	**0.922**<br>**<0.00001**	0.474<br>0.03488
**DTI-MD-Caudate**	-0.124<br>0.60236	-0.411<br>0.07160	-0.044<br>0.85515	0.236<br>0.31629	-0.205<br>0.39922	0.374<br>0.11503	-0.074<br>0.78016	0.161<br>0.49797	0.025<br>0.92050	**0.800**<br>**0.00002**	**0.502**<br>0.02402		**0.514**<br>0.02035	**0.523**<br>0.01789
**DTI-MD-Medulla**	-0.333<br>0.15080	-0.044<br>0.85240	-0.174<br>0.46201	0.398<br>0.08181	-0.279<br>0.24748	0.114<br>0.64204	-0.054<br>0.83888	-0.141<br>0.55221	0.167<br>0.49527	0.412<br>0.07105	**0.922**<br>**<0.00001**	**0.514**<br>0.02035		0.417<br>0.06771
**DTI-MD-Midbrain**	0.056<br>0.81336	-0.159<br>0.50328	0.319<br>0.17070	0.241<br>0.30686	-0.107<br>0.66279	0.177<br>0.46802	0.118<br>0.65265	0.227<br>0.33567	0.282<br>0.24133	0.388<br>0.09097	0.474<br>0.03488	**0.523**<br>0.01789	0.417<br>0.06771	

D

longitudinal Δ 3TD scanner	EDSS	SNRS	25FW	9HPT Avg	PASAT	MSFC	SDMT	CombiWISE	V-Ventricles	DTI-II-Caudate	DTI-T-Medulla	DTI-MD-Caudate	DTI-MD-Medulla	DTI-MD-Midbrain
**EDSS**		-0.372<br>0.00193	0.112<br>0.36603	-0.011<br>0.92934	-0.181<br>0.15171	-0.095<br>0.45539	-0.045<br>0.71763	**0.685**<br>**<0.00001**	-0.044<br>0.72698	0.165<br>0.20333	-0.194<br>0.13447	0.183<br>0.15714	-0.209<br>0.10564	-0.070<br>0.59169
**SNRS**	-0.372<br>0.00193		-0.185<br>0.13434	-0.046<br>0.71363	0.004<br>0.97433	-0.031<br>0.80784	-0.054<br>0.66442	**-0.782**<br>**<0.00001**	0.121<br>0.34031	0.005<br>0.96819	0.171<br>0.18749	-0.004<br>0.97546	0.196<br>0.13080	0.018<br>0.89052
**25FW**	0.112<br>0.36603	-0.185<br>0.13434		0.201<br>0.10245	-0.123<br>0.32910	0.442<br>**0.00023**	0.073<br>0.55560	0.486<br>**0.00003**	0.159<br>0.20633	0.096<br>0.45809	-0.060<br>0.64258	0.065<br>0.61740	-0.090<br>0.48506	-0.191<br>0.13678
**9HPT**	-0.011<br>0.92934	-0.046<br>0.71363	0.201<br>0.10245		0.038<br>0.76199	0.142<br>0.25798	-0.129<br>0.29699	0.180<br>0.14926	0.012<br>0.92504	-0.294<br>0.02138	0.060<br>0.64471	-0.234<br>0.07009	0.042<br>0.74553	0.085<br>0.51284
**PASAT**	-0.181<br>0.15171	0.004<br>0.97433	-0.123<br>0.32910	0.038<br>0.76199		-0.081<br>0.51986	0.177<br>0.15842	-0.125<br>0.32644	-0.026<br>0.83846	0.022<br>0.87013	0.249<br>0.05742	-0.010<br>0.94045	0.253<br>0.05331	0.060<br>0.64940
**MSFC**	-0.095<br>0.45539	-0.031<br>0.80784	0.442<br>**0.00023**	0.142<br>0.25798	-0.081<br>0.51986		-0.164<br>0.19246	0.128<br>0.31321	0.027<br>0.83669	0.184<br>0.16263	-0.073<br>0.58452	0.109<br>0.40994	-0.074<br>0.57606	-0.070<br>0.59945
**SDMT**	-0.045<br>0.71763	-0.054<br>0.66442	0.073<br>0.55560	-0.129<br>0.29699	0.177<br>0.15842	-0.164<br>0.19246		0.005<br>0.97040	-0.100<br>0.43014	0.116<br>0.37298	-0.079<br>0.54530	0.134<br>0.30161	-0.099<br>0.44988	-0.084<br>0.51770
**CombiWISE**	**0.685**<br>**<0.00001**	**-0.782**<br>**<0.00001**	0.486<br>**0.00003**	0.180<br>0.14926	-0.125<br>0.32644	0.128<br>0.31321	0.005<br>0.97040		-0.092<br>0.47365	0.158<br>0.22697	-0.177<br>0.17528	0.145<br>0.26978	-0.212<br>0.10345	-0.003<br>0.97983
**V-Ventricles**	-0.044<br>0.72698	0.121<br>0.34031	0.159<br>0.20633	0.012<br>0.92504	-0.026<br>0.83846	0.027<br>0.83669	-0.100<br>0.43014	-0.092<br>0.47365		-0.124<br>0.34774	-0.214<br>0.10387	-0.152<br>0.24967	-0.206<br>0.11769	-0.145<br>0.27475
**DTI-II-Caudate**	0.165<br>0.20333	0.005<br>0.96819	0.096<br>0.45809	-0.294<br>0.02138	0.022<br>0.87013	0.184<br>0.16263	0.116<br>0.37298	0.158<br>0.22697	-0.124<br>0.34774		0.069<br>0.59399	**0.950**<br>**<0.00001**	0.078<br>0.54814	0.313<br>0.01328
**DTI-T-Medulla**	-0.194<br>0.13447	0.171<br>0.18749	-0.060<br>0.64258	0.060<br>0.64471	0.249<br>0.05742	-0.073<br>0.58452	-0.079<br>0.54530	-0.177<br>0.17528	-0.214<br>0.10387	0.069<br>0.59399		0.090<br>0.48506	**0.965**<br>**<0.00001**	0.408<br>**0.00098**
**DTI-MD-Caudate**	0.183<br>0.15714	-0.004<br>0.97546	0.065<br>0.61740	-0.234<br>0.07009	-0.010<br>0.94045	0.109<br>0.40994	0.134<br>0.30161	0.145<br>0.26978	-0.152<br>0.24967	**0.950**<br>**<0.00001**	0.090<br>0.48506		0.095<br>0.46452	0.324<br>0.01020
**DTI-MD-Medulla**	-0.209<br>0.10564	0.196<br>0.13080	-0.090<br>0.48506	0.042<br>0.74553	0.253<br>0.05331	-0.074<br>0.57606	-0.099<br>0.44988	-0.212<br>0.10345	-0.206<br>0.11769	0.078<br>0.54814	**0.965**<br>**<0.00001**	0.095<br>0.46452		0.463<br>**0.00015**
**DTI-MD-Midbrain**	-0.070<br>0.59169	0.018<br>0.89052	-0.191<br>0.13678	0.085<br>0.51284	0.060<br>0.64940	-0.070<br>0.59945	-0.084<br>0.51770	-0.003<br>0.97983	-0.145<br>0.27475	0.313<br>0.01328	0.408<br>**0.00098**	0.324<br>0.01020	0.463<br>**0.00015**	

Figure S6: Correlations between validated MRI measures and clinical scales separated for two utilized 3T scanners

Correlation matrices for Mo -12 cross-sectional data **(A,B)** and relative percentage change (Δ) over one year **(C,D)** on 3TA **(A,C)** and 3TD **(B,D)** scanner in the progressive MS cohort. Each window shows the Spearman correlation coefficient and raw p value. P<0.0018 (the Bonferroni-adjusted significance level for adjusted for multiple comparisons of 28 tested variables) was considered as significant (yellow background). Significant correlation coefficients above 0.5 (blue) and below -0.5 (red) were considered biologically meaningful.

Figure S7

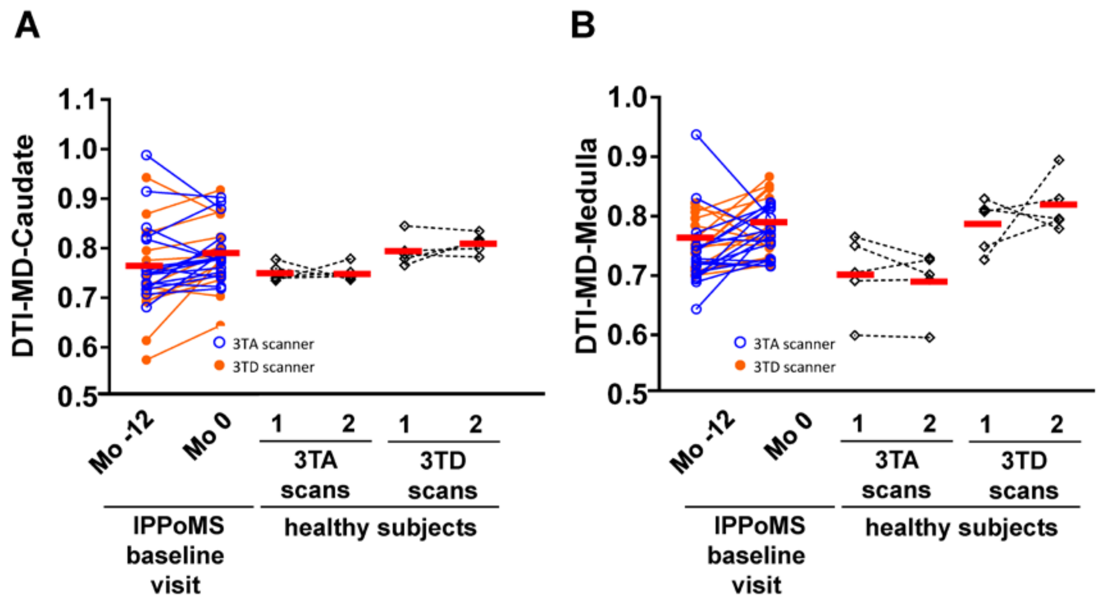

Figure S7: DTI measures with statistically significant percentage change over one year in three progressive MS cohorts show scanner effect and overlap with HV values

DTI mean diffusivity of the head of caudate **(A)** and the medulla **(B)** in IPPOMS1 cohort acquired over one-year baseline on 3TA (empty blue circles) and 3TD (full orange circles) scanners and scan-rescan variability for the same DTI measures in healthy volunteer cohort on both 3TA and 3TD scanner (empty black diamonds) show overlapping data between healthy volunteers and IPPOMS1 cohort, as well as a considerable scanner effect. The red bars represent the mean of each group.

Figure S8

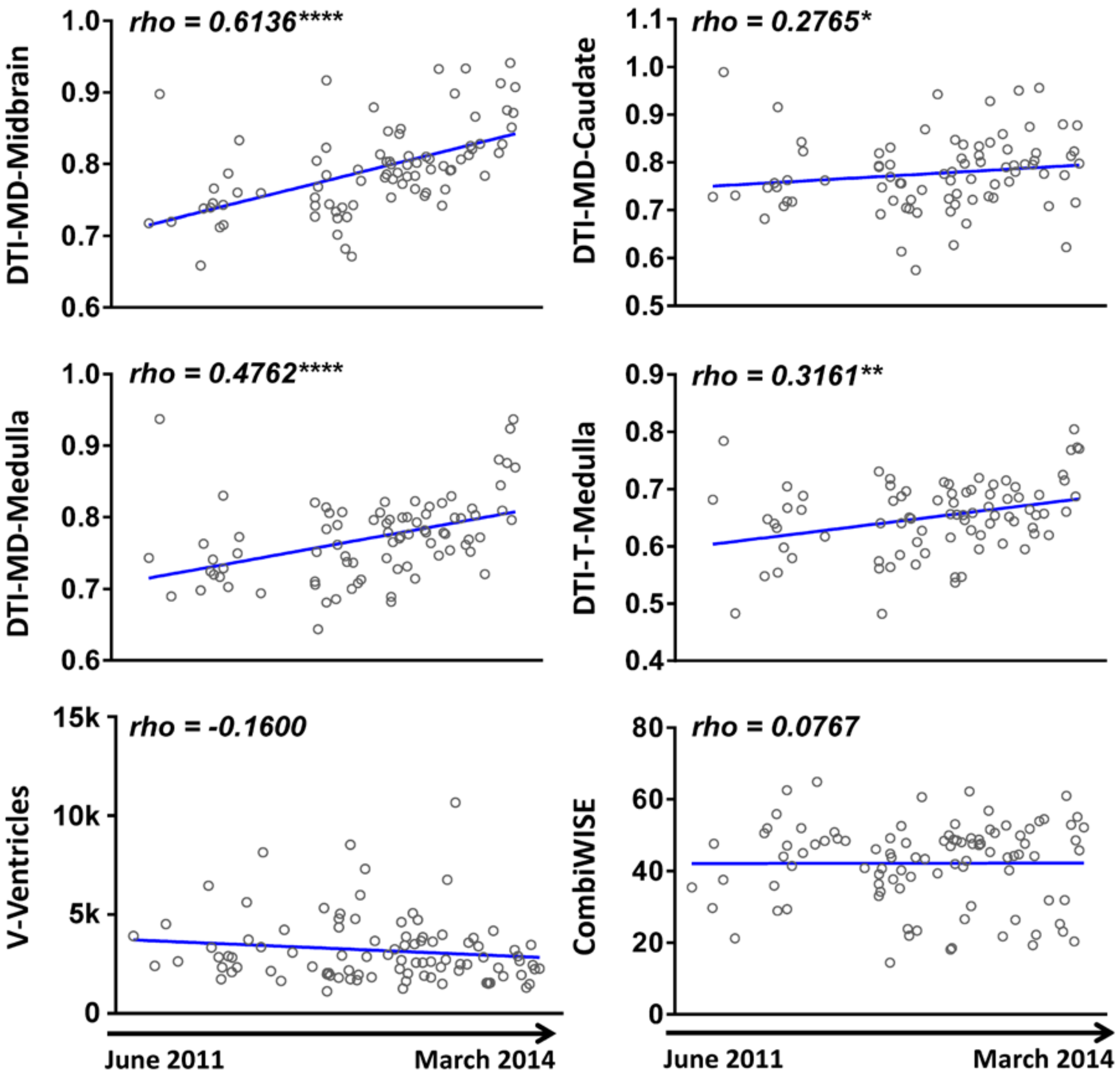

Figure S8: DTI measures show drift over time

Four validated DTI measures (mean diffusivity of the midbrain, caudate and medulla, radial diffusivity of the medulla) show statistically significant upward drift over the time period of nearly three years when – month -12 DTI measures are depicted as a function of MRI scan date. Validated volumetric MRI measure of the volume of ventricles, as well as CombiWISE do not show such a drift over the same period of time. Spearman correlation coefficients are shown; blue lines represent the best linear fit of the data points. * $p<0.05$, ** $p<0.01$, **** $p<0.0001$

Figure S9

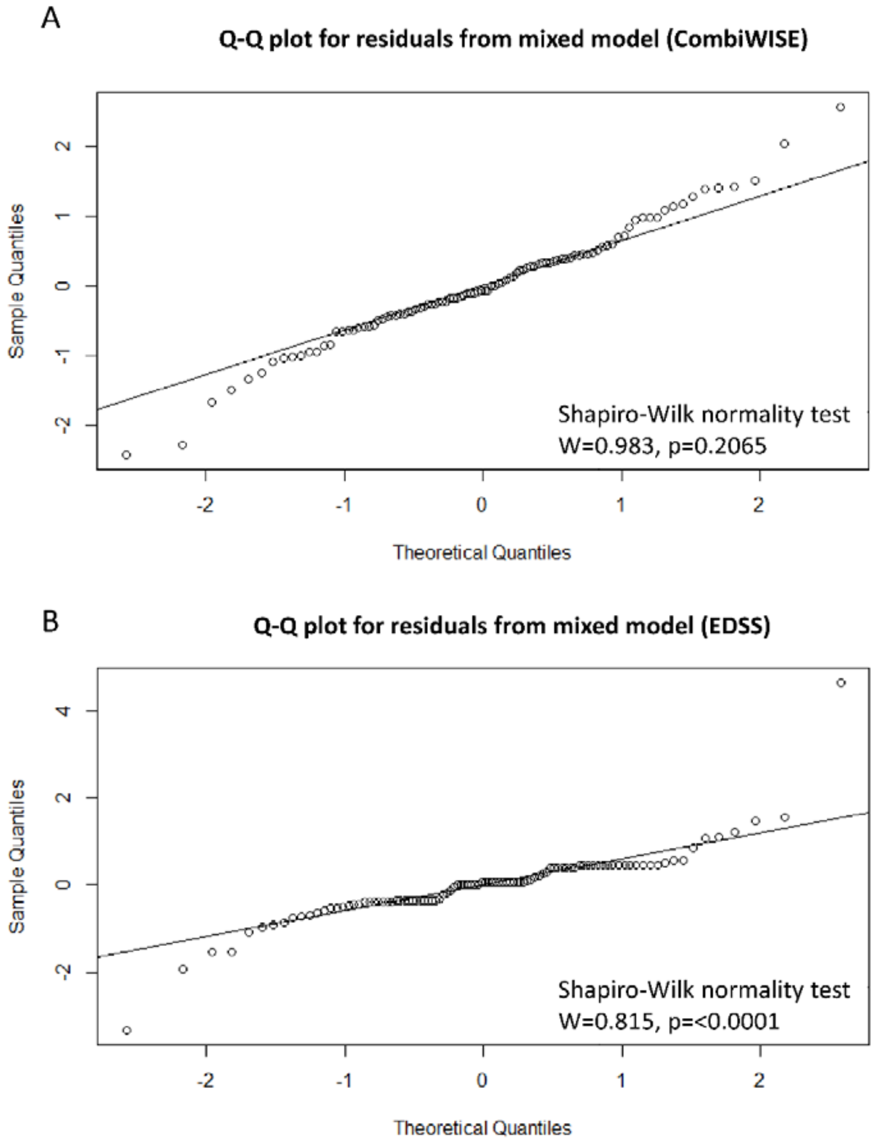

Figure S9: Behavior of residuals from mixed-models examining changes in CombiWISE and EDSS over time

QQ-plot of the Pearson residuals from mixed models predicting CombiWISE **(A)** and EDSS **(B)** using a linear change in time and a random intercept adjustment for each patient in the IPPOMS1 cohort. If the model residuals were normally distributed, the data points would be expected to fall close to the 1-1 line. The residuals from the model predicting CombiWISE **(A)** follow this line more closely than residuals from the model predicting EDSS **(B)**, indicating normally distributed residuals is a more reasonable assumption using CombiWISE than with EDSS. This is also confirmed using the Shapiro-Wilk test for normality on the residuals. Furthermore, the discrete nature of the EDSS scale can be observed as the residuals tend visually manifest as a step function rather than a continuous line.

Table S1: Calculation of CombiWISE based on EDSS, SNRS, 25FW, and 9HPT-NDH

(this is only a screenshot, please, refer to Excel file of Table S1 for a functional version)

EDSS values range from 0 to 10 with 0.5 unit change

SNRS values range from 100 to 0 with 1 unit change

25FW is performed in two trials, the value ranges between 0 and 179.9s. Patinets unable to complete the 25-foot walk in less than 3 min receive the maximum time value - 179.9

9HPT-NDH is performed in two trials, the value ranges between 0 and 300s. Patinets unable to complete the task in less than 5 min receive the value - 777

Patient Code	Date of Exam	EDSS	SNRS	25FW Time1	25FW Time2	9HPT-NDH Time1	9HPT-NDH Time2	CombiWISE
XX	12/1/2015	2.5	80	160	179.9	777	168	**38.94**
XY	12/2/2015	1	93	179.9	1	16.8	18.4	**22.60**
XZ	12/3/2015	6.5	54	16.5	18.2	90	102	**54.43**

Table S2: Spearman correlation coefficients, p-values, and number of observations measured in the cohort of 98 progressive MS patients in the cross-sectional comparisons at Mo -12 (above the diagonal) and longitudinal change over one year (below the diagonal).

month -12 / Δ over 12 months	EDSS	SNRS	25FW	9HPT	PASAT	MSFC	SDMT	CombiWISE	V-Ventricles	DTI-II-Caudate	DTI-T-Medulla	DTI-MD-Caudate	DTI-MD-Medulla	DTI-MD-Midbrain
**EDSS**		**-0.6935**<br>*<0.0001*<br>98	**0.8137**<br>*<0.0001*<br>98	**0.5264**<br>*<0.0001*<br>97	**-0.2183**<br>*0.0346*<br>94	**-0.6585**<br>*<0.0001*<br>94	**-0.3256**<br>*0.0014*<br>94	**0.8994**<br>*<0.0001*<br>97	**0.1607**<br>*0.1199*<br>95	**0.0301**<br>*0.7881*<br>82	**0.0838**<br>*0.4542*<br>82	**-0.0291**<br>*0.7954*<br>82	**-0.0059**<br>*0.9582*<br>82	**-0.0365**<br>*0.7450*<br>82
**SNRS**	**-0.2138**<br>*0.0355*<br>97		**-0.5602**<br>*<0.0001*<br>98	**-0.4807**<br>*<0.0001*<br>97	**0.2003**<br>*0.0529*<br>94	**0.5699**<br>*<0.0001*<br>94	**0.3640**<br>*0.0003*<br>94	**-0.8701**<br>*<0.0001*<br>97	**-0.0233**<br>*0.8226*<br>95	**-0.0007**<br>*0.9950*<br>82	**-0.0435**<br>*0.6982*<br>82	**-0.0053**<br>*0.9621*<br>82	**-0.0654**<br>*0.5592*<br>82	**-0.0671**<br>*0.5489*<br>82
**25FW**	**0.0760**<br>*0.4595*<br>97	**-0.2267**<br>*0.0256*<br>97		**0.5027**<br>*<0.0001*<br>97	**-0.1861**<br>*0.0725*<br>94	**-0.6879**<br>*<0.0001*<br>94	**-0.3064**<br>*0.0027*<br>94	**0.8362**<br>*<0.0001*<br>97	**0.1149**<br>*0.2673*<br>95	**-0.0102**<br>*0.9279*<br>82	**0.0048**<br>*0.9660*<br>82	**-0.0860**<br>*0.4423*<br>82	**-0.0416**<br>*0.7104*<br>82	**-0.0529**<br>*0.6367*<br>82
**9HPT**	**-0.0720**<br>*0.4858*<br>96	**-0.1419**<br>*0.1679*<br>96	**0.2221**<br>*0.0288*<br>97		**-0.2518**<br>*0.0144*<br>94	**-0.8426**<br>*<0.0001*<br>94	**-0.5444**<br>*<0.0001*<br>94	**0.6321**<br>*<0.0001*<br>97	**0.3079**<br>*0.0025*<br>94	**-0.1282**<br>*0.2542*<br>81	**-0.0267**<br>*0.8128*<br>81	**-0.1160**<br>*0.3023*<br>81	**-0.0332**<br>*0.7689*<br>81	**-0.1370**<br>*0.2225*<br>81
**PASAT**	**-0.2096**<br>*0.0437*<br>93	**0.1496**<br>*0.1524*<br>93	**-0.0971**<br>*0.3519*<br>94	**-0.0165**<br>*0.8744*<br>94		**0.5504**<br>*<0.0001*<br>94	**0.5365**<br>*<0.0001*<br>92	**-0.2335**<br>*0.0235*<br>94	**-0.3487**<br>*0.0007*<br>91	**-0.1157**<br>*0.3131*<br>78	**-0.2580**<br>*0.0226*<br>78	**-0.1325**<br>*0.2474*<br>78	**-0.2525**<br>*0.0258*<br>78	**-0.1558**<br>*0.1731*<br>78
**MSFC**	**-0.0550**<br>*0.6005*<br>93	**-0.0631**<br>*0.5480*<br>93	**0.3983**<br>*0.0001*<br>94	**0.1361**<br>*0.1910*<br>94	**-0.0759**<br>*0.4671*<br>94		**0.6068**<br>*<0.0001*<br>92	**-0.7503**<br>*<0.0001*<br>94	**-0.3529**<br>*0.0006*<br>91	**0.0759**<br>*0.5092*<br>78	**-0.1157**<br>*0.3130*<br>78	**0.0728**<br>*0.5267*<br>78	**-0.0737**<br>*0.5213*<br>78	**0.0184**<br>*0.8731*<br>78
**SDMT**	**0.0251**<br>*0.8121*<br>92	**0.0488**<br>*0.6439*<br>92	**-0.0557**<br>*0.5959*<br>93	**-0.2991**<br>*0.0036*<br>93	**0.1875**<br>*0.0751*<br>91	**-0.1520**<br>*0.1504*<br>91		**-0.4004**<br>*<0.0001*<br>94	**-0.4491**<br>*<0.0001*<br>91	**0.0813**<br>*0.4761*<br>79	**-0.0289**<br>*0.8007*<br>79	**0.1031**<br>*0.3658*<br>79	**-0.0458**<br>*0.6885*<br>79	**0.0503**<br>*0.6598*<br>79
**CombiWISE**	**0.6358**<br>*<0.0001*<br>96	**-0.7467**<br>*<0.0001*<br>96	**0.4953**<br>*<0.0001*<br>96	**0.1770**<br>*0.0844*<br>96	**-0.2055**<br>*0.0481*<br>93	**0.1328**<br>*0.2045*<br>93	**-0.0582**<br>*0.5813*<br>92		**0.1000**<br>*0.3377*<br>94	**-0.0121**<br>*0.9147*<br>81	**0.0599**<br>*0.5952*<br>81	**-0.0299**<br>*0.7909*<br>81	**0.0441**<br>*0.6958*<br>81	**-0.0037**<br>*0.9738*<br>81
**V-Ventricles**	**-0.0935**<br>*0.3753*<br>92	**0.0875**<br>*0.4069*<br>92	**0.1466**<br>*0.1607*<br>93	**-0.0319**<br>*0.7626*<br>92	**-0.0220**<br>*0.8380*<br>89	**0.0835**<br>*0.4363*<br>89	**-0.0329**<br>*0.7594*<br>89	**-0.0918**<br>*0.3870*<br>91		**-0.0565**<br>*0.6189*<br>80	**-0.0821**<br>*0.4689*<br>80	**-0.0660**<br>*0.5609*<br>80	**-0.0622**<br>*0.5835*<br>80	**-0.0521**<br>*0.6461*<br>80
**DTI-II-Caudate**	**-0.0210**<br>*0.8526*<br>81	**-0.1373**<br>*0.2216*<br>81	**0.0538**<br>*0.6315*<br>82	**-0.1706**<br>*0.1279*<br>81	**-0.0330**<br>*0.7742*<br>78	**0.1313**<br>*0.2518*<br>78	**0.0562**<br>*0.6249*<br>78	**0.1121**<br>*0.3220*<br>80	**-0.0775**<br>*0.4999*<br>78		**0.0853**<br>*0.4461*<br>82	**0.8910**<br>*<0.0001*<br>82	**0.0753**<br>*0.5016*<br>82	**0.3503**<br>*0.0013*<br>82
**DTI-T-Medulla**	**-0.1942**<br>*0.0824*<br>81	**0.0982**<br>*0.3832*<br>81	**-0.0979**<br>*0.3817*<br>82	**0.0787**<br>*0.4847*<br>81	**0.0791**<br>*0.4911*<br>78	**-0.0211**<br>*0.8548*<br>78	**-0.0061**<br>*0.9577*<br>78	**-0.1654**<br>*0.1426*<br>80	**-0.0856**<br>*0.4561*<br>78	**0.1357**<br>*0.2241*<br>82		**0.2118**<br>*0.0561*<br>82	**0.8995**<br>*<0.0001*<br>82	**0.3748**<br>*0.0005*<br>82
**DTI-MD-Caudate**	**0.0012**<br>*0.9917*<br>81	**-0.1220**<br>*0.2779*<br>81	**0.0212**<br>*0.8498*<br>82	**-0.1432**<br>*0.2023*<br>81	**-0.0479**<br>*0.6773*<br>78	**0.1430**<br>*0.2116*<br>78	**0.0964**<br>*0.4013*<br>78	**0.1050**<br>*0.3541*<br>80	**-0.0500**<br>*0.6640*<br>78	**0.9221**<br>*<0.0001*<br>82	**0.2006**<br>*0.0708*<br>82		**0.2459**<br>*0.0260*<br>82	**0.5329**<br>*<0.0001*<br>82
**DTI-MD-Medulla**	**-0.2829**<br>*0.0105*<br>81	**0.0634**<br>*0.5737*<br>81	**-0.1122**<br>*0.3155*<br>82	**0.0969**<br>*0.3893*<br>81	**0.0783**<br>*0.4954*<br>78	**-0.0153**<br>*0.8945*<br>78	**-0.0769**<br>*0.5034*<br>78	**-0.1963**<br>*0.0809*<br>80	**-0.0778**<br>*0.4984*<br>78	**0.1665**<br>*0.1349*<br>82	**0.9429**<br>*<0.0001*<br>82	**0.2257**<br>*0.0415*<br>82		**0.4628**<br>*<0.0001*<br>82
**DTI-MD-Midbrain**	**-0.1168**<br>*0.2990*<br>81	**-0.0643**<br>*0.5686*<br>81	**-0.0826**<br>*0.4607*<br>82	**0.1108**<br>*0.3249*<br>81	**0.0076**<br>*0.9471*<br>78	**-0.0245**<br>*0.8314*<br>78	**-0.0743**<br>*0.5180*<br>78	**0.0249**<br>*0.8262*<br>80	**-0.0501**<br>*0.6630*<br>78	**0.3780**<br>*0.0005*<br>82	**0.4127**<br>*0.0001*<br>82	**0.4066**<br>*0.0002*<br>82	**0.4619**<br>*<0.0001*<br>82	

Spearman rho
p-value
Number of Observations

Table S3: Yearly absolute change for selected MRI variables and scan-re-scan absolute change in healthy subjects

Biomarker abbreviation	Biomarker description	mean absolute difference over 12 months in in progressive MS patients[a]	SD	mean absolute difference of scan-re-scan in healthy subjects[b]	SD	signal-to-noise ratio[c]	
						one-year trial	two-year trial
V-SIENA	Change in brain volume (SIENA method)	1.111	1.112	0.199	0.243	5.578	11.156
V-Brain	Brain volume (Lesion Toads method)	11187	16062	3980	2888	2.811	5.621
V-Ventricles	Ventricular volume (Lesion Toads method)	1802	2181	632	558	2.851	5.703
V-CorticalGM	Volume of cortical gray matter (Lesion Toads)	17722	30598	15740	16506	1.126	2.252
V-Thalamus	Volume of thalamus (Lesion Toads)	520	743	490	420	1.063	2.126
V-Caudate+Putamen	Volume of caudate and putamen (Lesion Toads)	1022	1443	826	1126	1.238	2.476
qT1-IC	qT1 of anterior limb of internal capsule	72	54	130	86	0.556	1.112
qT1-CC	qT1 of corpus callosum	70	59	154	130	0.451	0.902
qT1-Caudate	qT1 of the head of the caudate nucleus	126	229	167	107	0.756	1.512
qT1-Putamen	qT1 of putamen	95	87	165	115	0.572	1.144
qT1-Thalamus	qT1 of thalamus	95	80	245	142	0.389	0.777
qT1-Midbrain	qT1 of midbrain (axial section)	83	87	200	137	0.416	0.831
qT1-Pons	qT1 of pons (sagittal section)	91	79	188	102	0.484	0.969
qT1-Medulla	qT1 of medulla (axial section)	108	96	214	159	0.501	1.003
DTI-T-IC	Radial diffusivity of internal capsule (anterior)	0.035	0.029	0.024	0.024	1.466	2.932
DTI-II-IC	Axial diffusivity of internal capsule (anterior)	0.089	0.083	0.066	0.064	1.334	2.669
DTI-MD-IC	Mean diffusivity of internal capsule (anterior)	0.046	0.036	0.032	0.022	1.465	2.931
DTI-FA-IC	Fractional anisotropy of internal capsule (anterior)	0.028	0.031	0.028	0.021	0.999	1.999
DTI-T-ICPost	Radial diffusivity of internal capsule (posterior)	0.038	0.028	0.017	0.017	2.242	4.483
DTI-II-ICPost	Axial diffusivity of internal capsule (posterior)	0.057	0.045	0.026	0.021	2.173	4.347
DTI-MD-ICPost	Mean diffusivity of internal capsule (posterior)	0.043	0.033	0.017	0.019	2.589	5.178
DTI-FA-ICPost	Fractional anisotropy of internal capsule	0.017	0.016	0.012	0.009	1.356	2.711

	(posterior)						
DTI-T-CC	Radial diffusivity of corpus callosum	0.039	0.031	0.018	0.016	2.119	4.238
DTI-II-CC	Axial diffusivity of corpus callosum	0.075	0.062	0.040	0.040	1.898	3.795
DTI-MD-CC	Mean diffusivity of corpus callosum	0.044	0.030	0.020	0.014	2.201	4.402
DTI-FA-CC	Fractional anisotropy of corpus callosum	0.024	0.022	0.014	0.014	1.733	3.465
DTI-T-Caudate	Radial diffusivity of the head of the caudate	0.054	0.041	0.017	0.012	3.194	6.387
DTI-II-Caudate	Axial diffusivity of the head of the caudate	0.067	0.051	0.046	0.039	1.446	2.893
DTI-MD-Caudate	Mean diffusivity of the head of the caudate	0.056	0.042	0.022	0.018	2.557	5.114
DTI-FA-Caudate	Fractional anisotropy of the head of the caudate	0.023	0.021	0.025	0.022	0.918	1.836
DTI-T-Putamen	Radial diffusivity of the putamen	0.044	0.031	0.021	0.015	2.086	4.172
DTI-II-Putamen	Axial diffusivity of the putamen	0.065	0.065	0.027	0.027	2.387	4.774
DTI-MD-Putamen	Mean diffusivity of the putamen	0.047	0.036	0.021	0.019	2.228	4.456
DTI-FA-Putamen	Fractional anisotropy of the putamen	0.025	0.027	0.016	0.013	1.545	3.089
DTI-T-Thalamus	Radial diffusivity of the thalamus	0.039	0.033	0.013	0.012	3.071	6.142
DTI-II-Thalamus	Axial diffusivity of the thalamus	0.051	0.042	0.024	0.015	2.121	4.241
DTI-MD-Thalamus	Mean diffusivity of the thalamus	0.041	0.034	0.014	0.010	2.898	5.796
DTI-FA-Thalamus	Fractional anisotropy of the thalamus	0.029	0.022	0.015	0.013	1.952	3.903
DTI-T-Midbrain	Radial diffusivity of the midbrain (axial section)	0.059	0.040	0.022	0.024	2.665	5.329
DTI-II-Midbrain	Axial diffusivity of the midbrain (axial section)	0.054	0.045	0.028	0.024	1.946	3.891
DTI-MD-Midbrain	Mean diffusivity of the midbrain (axial section)	0.054	0.039	0.018	0.022	2.939	5.879
DTI-FA-Midbrain	Fractional anisotropy of the midbrain (axial section)	0.033	0.031	0.020	0.014	1.685	3.371
DTI-T-Medulla	Radial diffusivity of the medulla (axial section)	0.042	0.036	0.054	0.055	0.764	1.527
DTI-II-Medulla	Axial diffusivity of the medulla (axial section)	0.050	0.059	0.034	0.031	1.498	2.997
DTI-MD-Medulla	Mean diffusivity of the medulla (axial section)	0.038	0.032	0.041	0.048	0.937	1.874

[a] calculated as an average of absolute difference between month -12 and month 0 in IPPOMS1,IPPOMS2 and RIVITALISE subjects
[b] calculated as an average of absolute difference between two scans on each scanner (3TA1 – 3TA2 and 3TD1 – 3TD2) for all healthy subjects
[b] signal-to-noise ratio for one year trial was calculated by dividing the mean change over 12 months in the progressive MS subjects by the mean of scan-re-scan difference of healthy subjects. For two-year trial the mean change in progressive MS patients was doubled (assuming a steady accumulation of disability in untreated subjects), while the mean scan-rescan change of healthy subjects remained unchanged.

Table S4: Number of required subjects per arm based on power analysis of seven statistically significant outcome measures.

Outcome Measure	***baseline vs. treatment design*** *(# of subjects per arm)*			***parallel group design*** *(# of subjects per arm)*		
	50% drug effect	*40% drug effect*	*30% drug effect*	*50% drug effect*	*40% drug effect*	*30% drug effect*
DTI-T Midbrain-MD	57	90	145	110	176	295
DTI-T Caudate-II	47	75	124	90	146	251
V-Ventricles	35	54	90	67	105	176
DTI-T Caudate-MD	30	47	79	57	90	155
DTI-T Medulla-MD	28	43	75	53	83	146
DTI-T Medulla-T	23	34	60	42	64	116
CombiWISE	19	28	49	34	53	95

Data S1: R codes

CombiWISE Development

```
library(tidyr)
library(dplyr)
library(beanplot)
library(nlme)
library(GA)
library(beanplot)
library(mosaic)
# Loads and cleans the data
#The dataset is contained in the “raw” R object, which is currently not provided
names(raw)<-c("patient","cohort","time","edss","snrs","walk1","walk2","walkavg",
"ndh1","ndh2","ndhavg","dh1","dh2","dhavg","hptavg","pasat","msfc",
"sdmt")
raw[,"WALK_FAIL"]<-ifelse(raw[,"walk1"]>=179.9|raw[,"walk2"]>=179.9,1,0)
raw[,"log2WALK_AVG"]<-ifelse(raw[,"WALK_FAIL"]==0,log2(raw[,"walkavg"]),0)
raw[,"NDH_FAIL"]<-ifelse(raw[,"ndh1"]==777|raw[,"ndh2"]==777,1,0)
raw[,"log2NDH_AVG"]<-ifelse(raw[,"NDH_FAIL"]==0,log2(raw[,"ndhavg"]),0)
raw[,"DH_FAIL"]<-ifelse(raw[,"dh1"]==777|raw[,"dh2"]==777,1,0)
raw[,"log2DH_AVG"]<-ifelse(raw[,"DH_FAIL"]==0,log2(raw[,"dhavg"]),0)
names(raw)<-toupper(names(raw))
# Calculate CombiWISE_V0
ippoms<-subset(raw,COHORT=="PPMS1" & TIME%in%c(-12,0))
names(ippoms)<-tolower(names(ippoms))
ippoms$ndh1<-with(ippoms,ifelse(ndh1==777,300,ndh1))
ippoms$ndh2<-with(ippoms,ifelse(ndh2==777,300,ndh2))
ippoms$dh1<-with(ippoms,ifelse(dh1==777,300,dh1))
ippoms$dh2<-with(ippoms,ifelse(dh2==777,300,dh2))
ippoms$ndhavg<-with(ippoms,(ndh1+ndh2)/2)
ippoms$dhavg<-with(ippoms,(dh1+dh2)/2)
ippoms$hptavg<-with(ippoms,(ndhavg+dhavg)/2)
ippoms$log2ndh_9hpt<-with(ippoms,log2(ndhavg))
ippoms$log2dh_9hpt<-with(ippoms,log2(dhavg))
ippoms$log29hpt<-with(ippoms,log2(hptavg))
ippoms$log2walk<-with(ippoms,log2(walkavg))
ippoms$patient<-as.factor(as.character(ippoms$patient))
```

```
ippoms$patient<-as.factor(as.character(ippoms$patient))
ippoms<-ippoms %>% tbl_df() %>% arrange(patient,time)
percent_change<-function(x){(((x[2]-x[1])/x[1])*100)}
zscore<-function(x){mean(x,na.rm=TRUE)/sd(x,na.rm=TRUE)}
new_weight<-ippoms %>% select(-cohort) %>% group_by(patient) %>%
summarize_each(funs(percent_change)) %>%
ungroup() %>% select(-patient,-time) %>% summarize_each(funs(zscore)) %>%
select(edss,snrs,log2walk,log29hpt) %>%
as.numeric()
a<-1/new_weight[1]
# Weights for CombiWISE_V0 Scale
c(100/10,100/100,100/log2(179.9),100/(2*log2(300)))*a*new_weight
## [1]    10.000000         -1.138207        14.481251        3.485333
raw$CombiWISE_V0<-with(raw,10*EDSS+1.138*(100-SNRS)+14.48*log2(WALKAVG)+3.49*(
log2((ifelse(raw$NDH1==777,300,raw$NDH1)+ifelse(raw$NDH2==777,300,raw$NDH2))/2)+
log2((ifelse(raw$DH1==777,300,raw$DH1)+ifelse(raw$DH2==777,300,raw$DH2))/2)))
raw<-raw[which(complete.cases(raw)==TRUE),]
# Patient level failure information using all scales
full<-raw[,c(1,3,4,5,20,19,22,21,24,23,16,18)]
raw$group<-with(raw,as.factor(as.character(interaction(WALK_FAIL,NDH_FAIL,DH_FAIL))))
pat<-raw %>% group_by(PATIENT) %>%
summarize(walk=as.numeric(sum(WALK_FAIL)>0),
ndh=as.numeric(sum(NDH_FAIL)>0),
dh=as.numeric(sum(DH_FAIL)>0)) %>%
mutate(group=as.factor(as.character(interaction(walk,ndh,dh))))
# group corresponds to the different failure indicators
# GA using all of the scales
# bounds on solution in the directions of disease progression
min<-c(0,-2,0,0,0,0,0,0,-2,-2);max<-min+2
# fitness function to optimize
fitness_lme<-function(beta,time=train$TIME,patient=train$PATIENT){
vec<-X%*%cbind(beta)
fit<-lme(vec~time,random=~1|patient)
return(out=summary(fit)$tTable[2,4]-as.numeric(beta[3]>beta[4])-
as.numeric(beta[5]>beta[6])-as.numeric(beta[7]>beta[8]))}
set.seed(239479)
```

```
nsims<-200
train_index<-matrix(0,ncol=69,nrow=nsims)
solutions<-matrix(0,ncol=10,nrow=nsims)
for(i in 1:nsims){
# Sampling 70% of patients stratified by failures to form training set
train_full<-pat %>% group_by(group) %>% sample_frac(0.7,replace=FALSE)
train<-full[full$PATIENT%in%train_full$PATIENT,]
train$PATIENT<-as.factor(as.character(train$PATIENT))
train_index[i,]<-which(pat$PATIENT%in%train$PATIENT)
X<-train[,-c(1,2)]
# Scaling by the maximum of the scale and running the GA
X<-scale(X,center=FALSE,scale=c(10,100,log2(179.9),1,log2(300),1,log2(300),1,60,110))
lmega<-ga(type="real-valued",fitness=fitness_lme,min=min,max=max,
crossover=gareal_blxCrossover,
names=colnames(X),maxiter=3000,run=400,
parallel=4,monitor=FALSE)
solutions[i,]<-lmega@solution[1,]
print(paste("Iteration",i,"Complete"))
}
# GA using the reduced set of scales
full<-raw[,c(1,3,4,5,20,19,22,21)]
raw$group<-with(raw,as.factor(as.character(interaction(WALK_FAIL,NDH_FAIL))))
# Patient level failures for reduced set of scales
pat<-raw %>% group_by(PATIENT) %>%
summarize(walk=as.numeric(sum(WALK_FAIL)>0),ndh=as.numeric(sum(NDH_FAIL)>0)) %>%
mutate(group=as.factor(as.character(interaction(walk,ndh)))) %>% data.frame()
min<-c(0,-2,0,0,0,0);max<-min+2
fitness_lme<-function(beta,time=train$TIME,patient=train$PATIENT){
vec<-X%*%cbind(beta)
fit<-lme(vec~time,random=~1|patient)
return(out=summary(fit)$tTable[2,4]-as.numeric(beta[3]>beta[4])-as.numeric(beta[5]>beta[6]))}
set.seed(239479) # seed used for the first 200 runs
set.seed(139479) # seed used for the next 300 runs
nsims<-200
train_index<-matrix(0,ncol=69,nrow=nsims)
solutions<-matrix(0,ncol=6,nrow=nsims)
```

```
for(i in 1:nsims){
train_full<-pat %>% group_by(group) %>% sample_frac(0.7,replace=FALSE)
train<-full[full$PATIENT%in%train_full$PATIENT,]
train$PATIENT<-as.factor(as.character(train$PATIENT))
train_index[i,]<-which(pat$PATIENT%in%train$PATIENT)
X<-train[,-c(1,2)]
X<-scale(X,center=FALSE,scale=c(10,100,log2(179.9),1,log2(300),1))
lmega<-ga(type="real-valued",fitness=fitness_lme,min=min,max=max,
crossover=gareal_blxCrossover,
names=colnames(X),maxiter=3000,run=400,
parallel=4,monitor=FALSE)
solutions[i,]<-lmega@solution[1,]
print(paste("Iteration",i,"Complete"))
}
# Solutions from GA
solutions_full<-read.csv("solutions_full.csv")
solutions_full<-solutions_full[,-1]
names(solutions_full)<-c("EDSS","SNRS","log2WALK_AVG","WALK_FAIL","log2NDH_AVG",
"NDH_FAIL","log2DH_AVG","DH_FAIL","PASAT","SDMT")
solutions_reduced<-read.csv("solutions_reduced.csv")
solutions_reduced<-solutions_reduced[,-1]
solutions_reduced2<-read.csv("solutions_reduced2.csv")
solutions_reduced2<-solutions_reduced2[,-1]
solutions_reduced<-rbind(solutions_reduced,solutions_reduced2)
names(solutions_reduced)<-c("EDSS","SNRS","log2WALK_AVG","WALK_FAIL","log2NDH_AVG",
"NDH_FAIL")
# Plots of Solutions from the GA for the full and reduced sets
beanplot(data.frame(solutions_full),col=c("beige",1,1,"red"),
log="",what=c(0,1,0,0),method="jitter",ylab="Relative Weights",
cex.axis=0.8,cutmin=-2,cutmax=2,main="Full Set")
abline(h=c(-2,0,2),col="grey",lty=2,lwd=2)
beanplot(data.frame(solutions_full),col=c("beige",1,1,"red"),
log="",what=c(0,0,1,1),method="jitter",add=T,
cex.axis=0.8,maxstripline=.05)
beanplot(data.frame(solutions_reduced),col=c("beige",1,1,"red"),
log="",what=c(0,1,0,0),method="jitter",ylab="Relative Weights",
```

```
cex.axis=0.8,cutmin=-2,cutmax=2,main="Reduced Set")
abline(h=c(-2,0,2),col="grey",lty=2,lwd=2)
beanplot(data.frame(solutions_reduced),col=c("beige",1,1,"red"),
log="",what=c(0,0,1,1),method="jitter",add=T,
cex.axis=0.8,maxstripline=.05)
# Rescaling of CombiWISE between 0 and 100
beta_mean<-apply(solutions_reduced,2,mean)
range<-c(10,100,log2(179.9),1,log2(300),1)
wei<-beta_mean/range # Weights divided by the ranges
dead<-c(10,0,0,1,0,1) # Theoretical worse possible measures
healthy<-c(0,100,log2(2),0,log2(15),0) # Theoretical best possible measures
# Note that this best possible measures assumes the walk is completed
# in 2 second on each trial and 9HPT completed in 15 seconds on
# each trial, which is improbable.
min<-wei%*%healthy # Theoretical Best Score
max<-wei%*%dead # Theoretical Worst Score
shift<-(-1*min) # Shift needed for min to be 0
scale<-(max+shift)/100 # Scale needed for max to be 100
int<-shift/scale # "Intercept Term"
final<-wei/scale # Weights to go with original Variables
comp_weight<-c(int,final)
names(comp_weight)<-c("Intercept",names(solutions_reduced))
beta_mean
##    EDSS         SNRS          log2WALK_AVG    WALK_FAIL    log2NDH_AVG
##    1.7668865    -1.8898529    0.8384609       0.8389831    0.4989071
##    NDH_FAIL
##    0.4995379
comp_weight
##    Intercept      EDSS          SNRS          log2WALK_AVG    WALK_FAIL
##    33.1661651     3.8026506     -0.4067296    2.4088947       18.0563935
##    log2NDH_AVG    NDH_FAIL
##    1.3048482      10.7509350
raw$CombiWISE<-
as.numeric(comp_weight[1]+as.matrix(raw[,c("EDSS","SNRS","LOG2WALK_AVG","WALK_FAIL",
"LOG2NDH_AVG","NDH_FAIL")])%*%cbind(comp_weight[-1]))
# Index of patients used to train each iteration
```

```
trainind<-read.csv("train_index_reduced.csv");
trainind<-trainind[,-1]
trainind2<-read.csv("train_index_reduced2.csv");
trainind2<-trainind2[,-1]
trainind<-rbind(trainind,trainind2)
# Scales for comparison with CombiWISE
raw$log2WALK<-with(raw,log2(WALKAVG))
raw$log2NDH_9HPT<-with(raw,log2(NDHAVG))
raw$log2DH_9HPT<-with(raw,log2(DHAVG))
raw$log29HPT<-with(raw,log2(HPTAVG))
# Validation of new scale using each iterations training and test sets
testlist<-as.list(names(raw)[c(27,4,5,28,29,30,31,16,17,18,25)])
validation_test<-validation_train<-matrix(0,nrow=500,ncol=length(testlist))
colnames(validation_train)<-colnames(validation_test)<-unlist(testlist)
for(i in 1:500){
pats_train<-as.character(pat[as.numeric(trainind[i,]),"PATIENT"])
pats_test<-as.character(pat[-as.numeric(trainind[i,]),"PATIENT"])
train<-raw[which(raw$PATIENT%in%pats_train),]
train$PATIENT<-as.factor(as.character(train$PATIENT))
test<-raw[which(raw$PATIENT%in%pats_test),]
test$PATIENT<-as.factor(as.character(test$PATIENT))
train_time<-train$TIME
train_patient<-train$PATIENT
validation_train[i,]<-unlist(lapply(testlist,function(ind){
metric<-train[,ind]
fit<-lme(metric~train_time,random=~1|train_patient)
return(summary(fit)$tTable[2,4])
}))
test_time<-test$TIME
test_patient<-test$PATIENT
validation_test[i,]<-unlist(lapply(testlist,function(ind){
metric<-test[,ind]
fit<-lme(metric~test_time,random=~1|test_patient)
return(summary(fit)$tTable[2,4])
}))
# print(paste("Iteration",i,"complete"))
```

```
}
validation_train[,3]<-abs(validation_train[,3])
validation_test[,3]<-abs(validation_test[,3])
colnames(validation_train)[3]<-colnames(validation_test)[3]<-"abs_SNRS"
colnames(validation_train)[7]<-colnames(validation_test)[7]<-"log2_9HPT"
# Test statistics from training and validation sets
beanplot(data.frame(validation_train[,-11]),col=c("beige",1,1,"red"),
log="",what=c(0,1,0,0),method="jitter",ylab="t-statistics",
main="Training Data Results",cex.axis=0.8)
abline(h=c(-2,0,2),col="grey",lty=2,lwd=2)
beanplot(data.frame(validation_train[,-11]),col=c("beige",1,1,"red"),
log="",what=c(0,0,1,1),method="jitter",add=T,
cex.axis=0.8,maxstripline=.05)
beanplot(data.frame(validation_test[,-11]),col=c("beige",1,1,"red"),
log="",what=c(0,1,0,0),method="jitter",ylab="t-statistics",
main="Validation Data Results",cex.axis=0.8)
abline(h=c(-2,0,2),col="grey",lty=2,lwd=2)
beanplot(data.frame(validation_test[,-11]),col=c("beige",1,1,"red"),
log="",what=c(0,0,1,1),method="jitter",add=T,
cex.axis=0.8,maxstripline=.05)
# Improvement upon SNRS
mean(validation_test[,"CombiWISE"]>validation_test[,"abs_SNRS"])
## [1] 0.934
mean(validation_test[,"CombiWISE"]-validation_test[,"abs_SNRS"])
## [1] 0.8473544
# Improvement upon EDSS
mean(validation_test[,"CombiWISE"]>validation_test[,"EDSS"])
## [1] 0.972
mean(validation_test[,"CombiWISE"]-validation_test[,"EDSS"])
## [1] 1.448859
# Validation of strength of correlation between CombiWISE and EDSS
names(cross)<-c("cohort","patient","edss","snrs","walk1","walk2","walkavg",
"ndh1","ndh2","ndhavg","dh1","dh2","dhavg","hptavg","pasat","msfc",
"sdmt")
cross<-cross[which(complete.cases(cross)==TRUE),]
cross[,"WALK_FAIL"]<-ifelse(cross[,"walk1"]>=179.9|cross[,"walk2"]>=179.9,1,0)
```

```
cross[,"log2WALK_AVG"]<-ifelse(cross[,"WALK_FAIL"]==0,log2(cross[,"walkavg"]),0)
cross[,"NDH_FAIL"]<-ifelse(cross[,"ndh1"]==777|cross[,"ndh2"]==777,1,0)
cross[,"log2NDH_AVG"]<-ifelse(cross[,"NDH_FAIL"]==0,log2(cross[,"ndhavg"]),0)
cross[,"DH_FAIL"]<-ifelse(cross[,"dh1"]==777|cross[,"dh2"]==777,1,0)
cross[,"log2DH_AVG"]<-ifelse(cross[,"DH_FAIL"]==0,log2(cross[,"dhavg"]),0)
names(cross)<-toupper(names(cross))
cross$CombiWISE<-as.numeric(comp_weight[1]+as.matrix(cross[
,c("EDSS","SNRS","LOG2WALK_AVG","WALK_FAIL","LOG2NDH_AVG","NDH_FAIL")])%*%
cbind(comp_weight[-1]))
# with(cross,cor(CombiWISE,EDSS,method="spearman"))
set.seed(934)
sims<-5000
null_cor<-numeric(0)
for(i in 1:sims){
cross_star<-cross
# Shuffles EDSS, recomputes CombiWISE using same weights, and
# tracks correlation.
cross_star$EDSS<-shuffle(cross_star$EDSS)
cross_star$CombiWISE<-as.numeric(comp_weight[1]+as.matrix(cross_star[
,c("EDSS","SNRS","LOG2WALK_AVG","WALK_FAIL","LOG2NDH_AVG","NDH_FAIL")])%*%
cbind(comp_weight[-1]))
null_cor[i]<-cor(cross_star$CombiWISE,cross_star$EDSS,method="spearman")
}
hist(null_cor,xlim=c(range(null_cor)[1],1),col="blue",main="Histogram of Expected Correlation",
xlab="Expected Correlation",nclass=30)
abline(v=with(cross,cor(CombiWISE,EDSS,method="spearman")),col="red",lwd=2)

# Graphical representation of increase in power
alpha<-0.05
df1<-1
df2<-70
grid_length<-1000
Fval<-seq(0,5,length=grid_length)^2
Fval1<-(seq(0,5,length=grid_length)+0.847)^2
Fval2<-(seq(0,5,length=grid_length)+1.449)^2
Fval3<-(seq(0,5,length=grid_length)+2)^2
```

```
Fcrit<-qf(1-alpha,df1=df1,df2=df2,ncp=0)
nc<-Fval*df1
nc1<-Fval1*df1
nc2<-Fval2*df1
nc3<-Fval3*df1
Fpow<-pf(Fcrit,df1=df1,df2=df2,ncp=nc,lower.tail=FALSE)
Fpow1<-pf(Fcrit,df1=df1,df2=df2,ncp=nc1,lower.tail=FALSE)
Fpow2<-pf(Fcrit,df1=df1,df2=df2,ncp=nc2,lower.tail=FALSE)
Fpow3<-pf(Fcrit,df1=df1,df2=df2,ncp=nc3,lower.tail=FALSE)
plot(Fpow~sqrt(Fval),ylab="Power",xlab="t-statistic",type="l",
main="Power Increase Using CombiWISE v. EDSS and Other Scales",xlim=c(0.5,5),lwd=4)
lines(sqrt(Fval),Fpow1,col=2,lty=2,lwd=4)
lines(sqrt(Fval),Fpow2,col=3,lty=3,lwd=4)
lines(sqrt(Fval),Fpow3,col=4,lty=4,lwd=4)
legend("bottomright",
legend=c("Baseline","0.847 unit increase from SNRS",
"1.449 unit increase from EDSS",
"2 unit increase from other scales"),
col=1:4,lty=1:4)

# Increase in CombiWISE per 1 unit increase in EDSS
summary(with(cross,lm(CombiWISE~EDSS)))$coefficients
##                Estimate    Std. Error    t value     Pr(>|t|)
##  (Intercept)   1.426832    0.3806608     3.748304    2.134254e-04
##  EDSS          7.495575    0.1003267 7   4.711656    2.372062e-196
```
